# Cost and cost-effectiveness of digital technologies for support of tuberculosis treatment adherence: a systematic review

**DOI:** 10.1136/bmjgh-2024-015654

**Published:** 2024-10-30

**Authors:** Cedric Kafie, Mona Salaheldin Mohamed, Miranda Zary, Chimweta Ian Chilala, Shruti Bahukudumbi, Genevieve Gore, Nicola Foster, Katherine L Fielding, Ramnath Subbaraman, Kevin Schwartzman

**Affiliations:** 1 McGill International Tuberculosis Centre, Research Institute of the McGill University Health Centre, Montreal, Quebec, Canada; 2 TB Centre, London School of Hygiene and Tropical Medicine, London, UK; 3 Department of Public Health and Community Medicine, Tufts University School of Medicine, Boston, Massachusetts, USA; 4 McGill Schulich Library of Physical Sciences, Life Sciences and Engineering, McGill University, Montreal, Quebec, Canada; 5 Division of Geographic Medicine and Infectious Diseases, Tufts Medical Center, Boston, Massachusetts, USA

**Keywords:** Tuberculosis, Treatment, Systematic review, Global Health

## Abstract

**Background:**

Digital adherence technologies (DATs) may provide a patient-centred approach to supporting tuberculosis (TB) medication adherence and improving treatment outcomes. We synthesised evidence addressing costs and cost-effectiveness of DATs to support TB treatment.

**Methods:**

A systematic review (PROSPERO-CRD42022313531) identified relevant literature from January 2000 to April 2023 in MEDLINE, Embase, CENTRAL, CINAHL, Web of Science along with preprints from medRxiv, Europe PMC and ClinicalTrials.gov. Studies with observational, experimental or quasi-experimental designs (minimum 20 participants) and modelling studies reporting quantitative data on the cost or cost-effectiveness of DATs for TB infection or disease treatment were included. Study characteristics, cost and cost-effectiveness outcomes were extracted.

**Results:**

Of 3619 titles identified by our systematic search, 29 studies met inclusion criteria, of which 9 addressed cost-effectiveness. DATs included short message service (SMS) reminders, phone-based technologies, digital pillboxes, ingestible sensors and video-observed therapy (VOT). VOT was the most extensively studied (16 studies) and was generally cost saving when compared with healthcare provider directly observed therapy (DOT), particularly when costs to patients were included—though findings were largely from high-income countries. Cost-effectiveness findings were highly variable, ranging from no clinical effect in one study (SMS), to greater effectiveness with concurrent cost savings (VOT) in others. Only eight studies adequately reported at least 80% of the elements required by Consolidated Health Economic Evaluation Reporting Standards, a standard reporting checklist for health economic evaluations.

**Conclusion:**

DATs may be cost saving or cost-effective compared with healthcare provider DOT, particularly in high-income settings. However, more data of higher quality are needed, notably in lower-income and middle-income countries which have the greatest TB burden.

**WHAT IS ALREADY KNOWN ON THIS TOPIC**

- Digital adherence technologies (DATs) can provide a less intrusive, and potentially less resource-intensive way to monitor and support tuberculosis (TB) treatment adherence, as compared with traditional direct observation. To date, there is limited information about the cost and cost-effectiveness of these technologies in diverse care settings.

**WHAT THIS STUDY ADDS**

- Our comprehensive review of available studies shows that some DATs like video-observed therapy can be cost saving, particularly in higher-income countries, and especially when patient costs are considered.

**HOW THIS STUDY MIGHT AFFECT RESEARCH, PRACTICE OR POLICY**

- While programme savings related to some DATs will likely offset their initial costs in higher-income settings, more evidence is needed from lower-income settings where the TB burden is highest. Costing studies should also more rigorously account for all relevant costs, including those to patients.

## Background

 One-quarter of the world’s population is believed to have been infected with *Mycobacterium tuberculosis*.^1^ In 2022, an estimated 10.6 million people fell ill with tuberculosis (TB) disease worldwide and a total of 1.3 million people died from TB.^2^

Treatment for TB disease typically involves multiple antibiotics for at least 6 months, with a 4-month regimen recently introduced in some settings.^3^ Poor adherence to anti-TB treatment may lead to treatment failure and relapse. Directly observed therapy (DOT), where an observer witnesses all or most doses, is commonly instituted with the goal of improving adherence but is seen as both coercive and resource-intensive.^4^ Particularly when administered by healthcare providers in a clinic, DOT often requires frequent travel, time off work and additional childcare expenses, which can place a large financial and emotional burden on persons with TB.

TB infection (TBI), the state of infection with *Mycobacterium tuberculosis* without any clinical symptoms, radiographic progression or detectable bacteria, is usually treated for 3–9 months with one or more anti-TB antibiotics to clear the infection and prevent the development of active disease. Suboptimal adherence substantially limits the individual and population health benefits of such preventive treatment.

Digital adherence technologies (DATs) may facilitate more patient-centric approaches for monitoring TB medication adherence than existing DOT models.^5^ DAT interventions include smartphone-based technologies, short message service (SMS) or video-supported treatment, digital pillboxes and ingestible sensors that aim to monitor and improve adherence to TB treatment.

Technologies that reduce travel and time requirements could produce significant cost savings while improving the treatment experience for persons with TB. However, these devices can carry significant technology costs, which are a particular challenge in lower-income settings. In addition, their impact on clinical outcomes varies by technology, intervention approach and setting. Depending on the setting and care model, some reports have suggested that DAT-based treatment may be cost-effective or even cost-saving relative to the standard of care (which often varies), but these findings have been inconsistent.^6^

We conducted a systematic review, to summarise existing evidence addressing the cost and cost-effectiveness of DATs for TB disease and TBI.

## Methods

The protocol for this systematic review was registered in PROSPERO, the International Prospective Register of Systematic Reviews (CRD42022313531)^7^ and is summarised below.

### Search and screening strategy

The search was conducted on 25 April 2023 (updated from 14 April 2022) and included reports published in MEDLINE/Ovid, Embase/Ovid, CENTRAL/Wiley, CINAHL and Web of Science Core Collection, from 1 January 2000 to 14 April 2023. We also searched Europe PMC for preprints (including medRxiv) and ClinicalTrials.gov for unpublished clinical trials and investigators of interest. Key search terms related to TB (active or latent), digital technologies (such as mobile phones, smartphones, video observation, medication monitors and text messaging) and cost (such as cost, economic cost-effectiveness, incremental cost-effectiveness ratio). A complete list of search terms can be found in online supplemental section S1. The database search was conducted by a health librarian (GG). Separately, a handsearch was conducted through the Union World Conference on Lung Health conference proceedings for relevant abstracts on DATs and costs from 2004 to 2022 inclusively. There were no language restrictions.

### Inclusion/exclusion criteria

Studies were included if they reported quantitative cost, budget impact or cost-effectiveness estimates for the use of DATs for TB treatment support (eg, pure cost descriptions, incremental cost comparisons and cost per health gain). The minimum number of participants required to use the DAT was 20 except for modelling studies, which typically reflected hypothetical cohorts and cost inputs from a variety of sources. DAT interventions included but were not limited to smartphone-based technologies, SMS or video-supported treatment, digital pillboxes and ingestible sensors. Studies had to involve DAT use to support treatment adherence in individuals diagnosed and treated for TB disease or infection, including persons generally at higher risk of unfavourable outcomes (eg, those with drug-resistant TB, persons living with HIV). A full case definition for DATs is included in online supplemental section S2.

Eligible study designs included randomised controlled trials, quasi-experimental trials, observational studies and modelling studies. Articles were excluded if the technology was not used to improve TB treatment adherence; we also excluded review articles, editorials, commentaries, news articles and protocols, as well as abstracts other than those from the Union World Conference on Lung Health. Relevant grey literature publications (such as preprints, ministry reports and technical papers) were eligible if they met all inclusion criteria.

### Study selection

After deduplication using EndNote,^8^ each title and abstract was screened independently (blinded) by at least two reviewers (among CK, MSM, MZ, CIC and SB) using Rayyan.ai^9^ to establish whether the publication in question potentially addressed DATs for TB treatment support and potentially met the other inclusion criteria. Studies for possible inclusion then underwent independent full-text review by two reviewers, for eligibility according to the detailed criteria above. Conflicts during each stage were resolved by consensus, with inclusion of a third senior reviewer when needed (KLF, RS and KS). All publications cited by the included articles and all publications that cited them (using Google Scholar^10^) were also screened for inclusion.

### Data extraction

After identifying all eligible studies, their data were extracted into a standard template in Excel^11^ by two independent reviewers (among CK, MSM and MZ) and subsequently compared. Any conflicts were resolved by consensus and by discussion with a third reviewer when necessary. Extracted data included detailed information on the study characteristics, study design, study setting (inpatient or outpatient), participant characteristics, DAT used, intervention duration, standard of care (comparator) and any important gaps noted by the reviewers. The total costs (for the DAT and any comparator) and cost-effectiveness estimates provided by the reports were extracted, as was an inventory of the cost components included in the total (eg, equipment and personnel time). Online supplemental table A1 lists and describes the types of costs tabulated for each study, whenever available. Studies that reported multiple sensitivity analyses and scale-up scenarios were noted but only the base case results were extracted.

We also extracted all available information on the scope, frequency (eg, daily) and mode (eg, self-administered therapy (SAT) or DOT) of the comparator. Whenever DOT was used as a comparator for a digital technology, we also noted the method of delivery (eg, clinic or field based). While specifics varied, we noted three main models for DOT delivery. Clinic DOT required the person taking TB treatment to attend a central facility such as a clinic, a hospital or a prison for observation. Field DOT required health workers to travel to the person’s home, workplace or other community location for dosing observation. Family DOT allowed observation by designated treatment supporters among family members or close friends. We noted studies where the mode for DOT was not further characterised as ‘DOT—not reported’, that is, DOT-NR.

### Data synthesis

In our primary analysis, we expressed costs in ‘international’ dollars based on purchasing power parity (PPP), reflecting equivalent purchasing power in each study setting to that provided by a US dollar (USD) in the USA.^12^ This enhances comparability across diverse settings and attenuates distortion from abrupt fluctuations in market exchange rates (eg, if a currency is revalued).^13^ It is particularly relevant for local decision-makers and funders, as opposed to international donors. Strictly speaking, the use of PPP may be best suited for non-tradable goods, for example, local labour. However, in DAT cost studies, labour and domestic transport are often the major component and a detailed breakdown between tradable and non-tradable goods may not be available.

Hence costs from countries other than the USA were first inflated to 2022 costs using the local gross domestic product (GDP) implicit price deflator as recommended for non-traded goods^13 14^ and then converted to international dollars using PPP estimates reported by the International Monetary Fund (IMF).^15^ US-based costs were inflated to 2022 dollars using the US GDP deflator from the same IMF dataset.^15^

We also performed a sensitivity analysis where all costs were converted to USD using average annual market exchange rates from the IMF.^16^ When not otherwise reported, the currency year was assumed to be the publication year, and the average market exchange rates for that year were used for currency conversion. A list of all factors used for cost adjustment is provided in online supplemental table A2.

We grouped cost results by DAT and tabulated the costs per person treated along with key details (eg, country, number of participants, comparator and cost components). Cost outcomes were further grouped by the costing perspective (ie, only costs borne by healthcare providers or societal costs which also include costs to patients and family members). For video-observed therapy (VOT) studies that only reported costs per treatment observation, costs were converted to a standard 6-month (26-week) treatment regimen with VOT performed 7X/week and DOT performed 5X/week to facilitate comparison: these were the most common observation frequencies for VOT and DOT, respectively. For studies comparing VOT to DOT, we also present the cost per observation for all studies that reported scheduled observation frequencies.

When costs were available for multiple sites in a single country, only the weighted mean of all sites was presented, with weights reflecting the number of participants at each site. For such studies, information from individual sites is reported in online supplemental table A4. Unless otherwise specified, reported costs for TB disease reflect drug-sensitive disease. Cost-effectiveness results were grouped by outcome type (eg, cost per disability-adjusted life-year (DALY) averted, cost per quality-adjusted life-year (QALY) gained) and by costing perspective.

### Quality assessment

Quality of reporting was evaluated using the Consolidated Health Economic Evaluation Reporting Standards (CHEERS) 2022 checklist^17^ for full-text studies only. The checklist includes 28 criteria. A score of 1 was assigned for each criterion when fully met, 0 when it was not, and NA when it was not applicable. For each study, we calculated the percentage of checklist criteria met, after exclusion of those which were not applicable. This quality assessment was performed independently by two reviewers. Any differences between the two reviewers were resolved by consensus, with discussion with a third reviewer when necessary. Certainty of evidence was not rated formally but was discussed narratively.

### Patient and public involvement

Patients and the public were not specifically involved in the design, conduct, reporting or dissemination plans of our research.

## Results

### Search results

**Figure 1:**
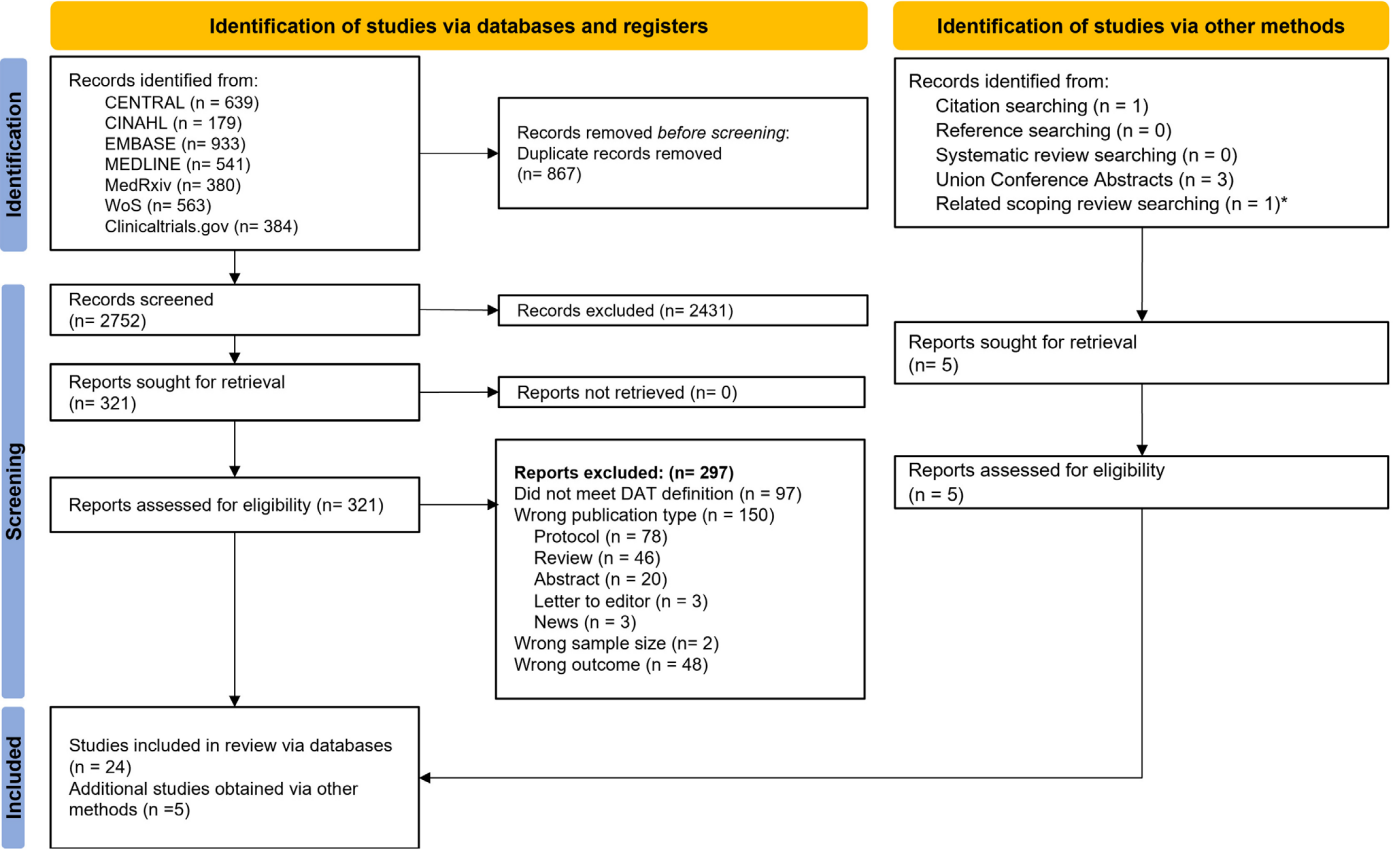
PRISMA flow diagram. *This study was captured by a related scoping review search by the authors of this systematic review and met all inclusion criteria. DAT, digital adherence technology; PRISMA, Preferred Reporting Items for Systematic Reviews and Meta-Analyses.

Of the 3619 records identified by the initial search, 867 were removed as duplicates and 2752 titles and abstracts were screened. Of these, 321 addressed DATs and TB and underwent full-text review for eligibility. Of these, 24 met inclusion criteria, while 5 others were identified by supplementary search of references and citations, for a total of 29 studies included in our review. Figure 1 shows the Preferred Reporting Items for Systematic Reviews and Meta-Analyses 2020 flow chart for included and excluded studies.

### Study characteristics

**Figure 2:**
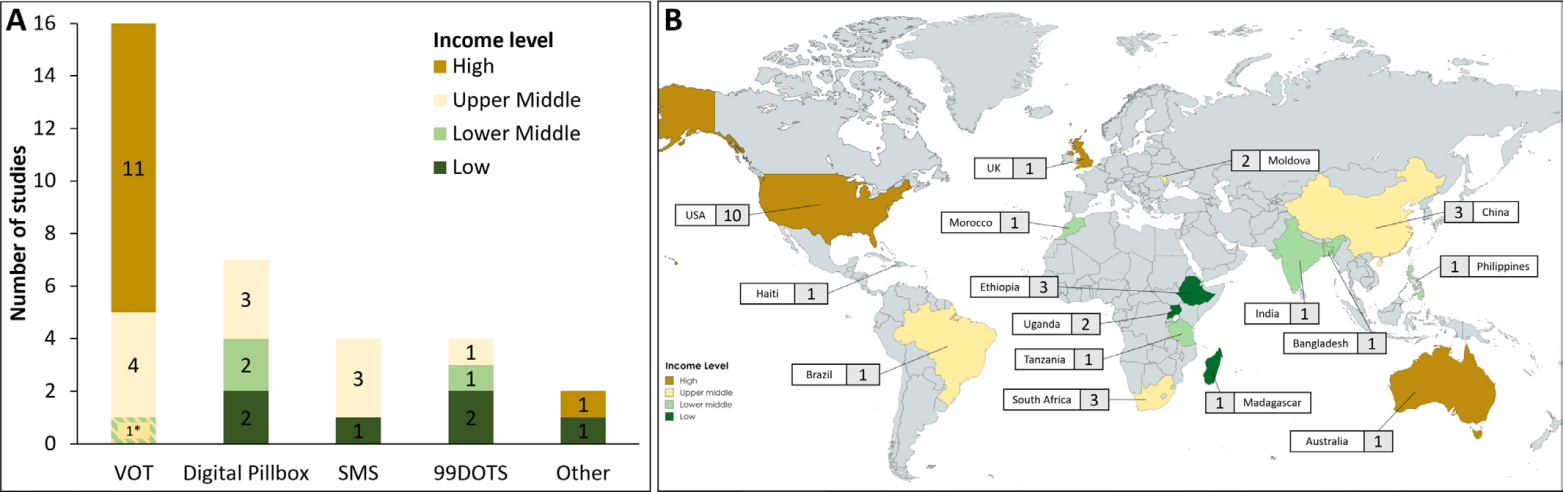
Summary of included studies. (**A**) Number of studies evaluating each DAT type by country income level. *One study analysed VOT in lower-middle and upper-middle-income countries. Nsengiyumva *et al*,^19^ and Nsengiyumva *et al*,^20^ analysed multiple DAT types (see table 1) and thus are included in multiple columns above. (**B**) Map of countries coloured by income level and number of included studies from each. DAT, digital adherence technology; DOT, directly observed therapy. SMS, short message service

Detailed characteristics of each included study are listed in table 1, while figure 2 highlights the DAT types and country settings by income level. Over half the included studies evaluated VOT (16 studies), followed by digital pillboxes (7 studies), SMS (4 studies) and medication sleeves with phone calls (‘99DOTS’; 4 studies). There were two other DAT interventions addressed by one study each: automated interactive voice calls and ingestible sensors.

**Table 1:** Characteristics of included studies

Authors	Year	Study design	DAT	Comparator(s)	Country	Effectiveness
Au-Yeung and DiCarlo^30^	2012	Modelling	Ingestible sensors—record doses ingested via a wearable device	DOT—clinic	USA****	
Bahrainwala *et al*^40^	2020	Modelling	Multicomponent intervention—only Digital pillbox^§^ component extracted	2 months DOT+4 months SAT and SAT alone	Madagascar*	✓
Beeler Asay *et al*^34^	2020	Observational	VOT—synchronous and asynchronous	DOT—clinic and field	USA****	
Broomhead and Mars^38^	2012	Observational	Digital pillbox—prompts patient via SMS when doses are missed	DOT—NR	S. Africa***	
Buchman and Cabello^46^	2017	Observational	VOT—synchronous	DOT—field	USA****	
Daftary *et al*^31^	2017	Experimental	Interactive automated voice calls—daily reminders to take medication, reminders for clinic visits, and monthly adherence and side effect assessments	SAT (not costed)	Ethiopia*	
Fekadu *et al*^47^	2021	Modelling	VOT—synchronous	SAT and DOT (clinic)	USA****	✓
Garfein *et al*^41^	2018	Observational	VOT—asynchronous with daily SMS or email reminders	DOT—Field^+^	USA****	
Gashu *et al*^42^—preprint	2021	Experimental	SMS reminders—(daily) include graphical messages for illiterate patients	DOT (not costed)	Ethiopia*	
Guo *et al*^48^ (A)	2020	Experimental	VOT—synchronous	DOT—NR	China***	
Guo *et al*^18^ (B)	2020	Observational	VOT—asynchronous with automated reminders in case of missed recordings	DOT—clinic	China***	
Holzman *et al*^37^	2018	Observational	VOT—asynchronous with automated reminders in case of missed recordings	DOT—field	USA****	
Krueger *et al*^23^	2010	Observational	VOT—synchronous	DOT—field	USA****	
Lam *et al*^35^	2019	Observational	VOT—synchronous and asynchronous	DOT—clinic and field	USA****	
Louwagie *et al*^27^	2022	Experimental	SMS reminders—twice weekly (10 TB-related and 7 smoking or alcohol reduction related)+3 motivational counselling sessions	Not explicitly described—likely SAT based on costs included	S. Africa***	✓
Manyazewal *et al*^25^	2022	Experimental	Digital pillbox—data downloaded at follow-up visits and discussed with patients	DOT—clinic	Ethiopia*	
Mukora *et al*^49^—abstract	2022	Experimental	Digital pillbox—triggers SMS, phone calls and home visits in cases of non-adherence	SAT	S. Africa***	
Nsengiyumva *et al*^19^	2018	Modelling	VOT—synchronous, Digital pillbox with SMS reminder in case of missed dose, two-way SMS, phone calls with medication sleeves—branded 99DOTs	SAT and DOT (clinic)	Brazil***	✓
Nsengiyumva *et al*^20^—preprint	2023	Observational	VOT asynchronous, phone calls with medication sleeves—branded 99DOTs	DOT—field (not costed), clinic and family	Tanzania** Moldova*** Philippines** Bangladesh** Haiti**	
Peng *et al*^26^—abstract	2014	Experimental	SMS reminders—no further details are provided in abstract	DOT—NR	China***	
Ravenscroft *et al*^24^	2020	Experimental	VOT—asynchronous	DOT—clinic	Moldova***	
Saha *et al*^33^	2022	Quasi-experimental	Digital pillbox—reminds patients daily+provides real-time monitoring on app for healthcare workers	NR—but referenced DOT costs from literature	India**	✓
Salcedo *et al*^32^	2021	Modelling	VOT—asynchronous—videos screened first by AI software (branded AiCure)	DOT—combined field (69% of doses) and clinic (31%)	USA****	✓
Siddiqui *et al*^36^	2019	Observational	VOT—asynchronous	DOT—field	USA****	
Story *et al*^21^	2019	Experimental	VOT—asynchronous	DOT—clinic	UK****	
Thompson *et al*^29^	2022	Experimental	Phone calls with medication sleeves—branded 99DOTs	DOT—family	Uganda*	✓
Wade *et al*^22^	2012	Observational	VOT—synchronous (with 5% of patients remaining on DOT)	DOT—field	Australia****	✓
Waswa *et al*^28^—abstract	2022	Experimental	Phone calls with medication sleeves—branded 99DOTs	None costed	Uganda*	
Yang *et al*^39^	2022	Modelling^¶^	Digital pillbox—prompts healthcare workers if dose is missed to facilitate follow-up	2 months DOT—NR+4 months SAT	Morocco****	✓

Country income level (2021) from World Bank: *low, **lower middle, ***upper middle, ****high.

+Mostly field DOT but a small unspecified number of patients were on clinic DOT in one of the clinics studied.

¶Also contained cost information from retrospective cohort study by Park *et al*.^50^

§This study evaluated a multicomponent intervention labelled drone-observed therapy system which included the use of digital pillboxes. Importantly, only the cost of the isolated digital pillbox was extracted which was available from sensitivity analyses.

DOT, directly observed therapy; NR, not reported; SAT, self-administered therapy; SMS, short message service; VOT, video-observed therapy.

The scope of included costs varied widely among the 26 full-text studies (online supplemental table A3). For example, a few studies included up-front implementation costs, such as staff training and programme setup; most did not. Only six considered costs borne by persons with TB or their families, four of which also considered indirect costs such as lost wages. Eight studies included the cost of TB treatment, such as TB medication and follow-up testing. This was generally to model downstream cost savings associated with potential improvements in health outcomes. For example, if the intervention was thought to reduce acquired drug resistance, then the intervention would also reduce the additional treatment costs associated with multidrug-resistant TB disease (MDR-TB). However, most studies did not consider such second-order effects.

### Video-observed therapy

Reported provider costs for VOT are summarised in table 2. There was enormous variation, ranging from international $9 per person treated in China, when only transport costs were considered^18^ to international $21 817 in a Brazil modelling study^19^ which considered all costs including medications and follow-up testing for persons with MDR-TB. The median estimated provider cost across 15 reports was $1364 per patient. For studies that reported costs for multiple sites in a single country, only the weighted average cost per patient is shown. The results for individual sites are provided in online supplemental table A4. Three studies also considered scale-up scenarios^20^,^22^ with larger hypothetical patient numbers that are not presented here. One analysis only considered averted DOT costs (such as fuel and labour)^23^ but excluded any additional costs associated with VOT, so cost savings were inevitable and not necessarily reflective of all potential costs.

**Table 2:** Provider and societal costs for video-observed therapy (VOT) and standard of care (SoC) in 2022 international dollars

Study	Country	DAT pts.	VOT Type	Duration	SOC	Provider costs per person treated			Costs included
						VOT	SoC	Incremental	
Beeler Asay *et al*^34^ (2020)	USA	63	Async.	6 months - Calc.	DOT (field)	$2471	$2888	–$417	S, T, F, I
				6 months - Calc.	DOT (clinic)	$2471	$1856	$616	
		50	Sync.	6 months - Calc.	DOT (field)	$1208	$2888	–$1680	
				6 months - Calc.	DOT (clinic)	$1208	$1856	–$648	
Buchman and Cabello^46^ (2017)	USA	24	Sync.	NR	DOT (field)	Incremental		–$1256	S, T, F
Fekadu *et al*^47^ (2021)	USA	NR*	Sync.	6 months	SAT	$13 428	$15 353	–$1925	S, D, T, F
				6 months	DOT (clinic)	$13 428	$15 048	–$1620	
Garfein *et al*^41^ (2018)	USA	225	Async.	6 months	DOT (field)	$4108	$5549	–$1441	S, T, F
Guo *et al*^48^ (A) (2020)	China	199	Sync.	NR	DOT (NR)	$9	$19	–$10	S, T, F
Guo *et al*^18^ (B) (2020)	China	90	Async.	6 months	DOT (clinic)	$14	$73	–$59	T (Transport only†)
Holzman *et al*^37^ (2018)	USA	15/vehicle‡	Async.	6 months	DOT (field)	$775	$2373	–$1599	S, T, F
Krueger *et al*^23^ (2010)	USA	57	Sync.	5 months	DOT (field)	Incremental		–$3228	S, T
Lam *et al*^35^ (2019)	USA	81	Sync.	6 months - Calc.	DOT (clinic)	$1427	$1319	$108	S, T, F
				6 months - Calc.	DOT (field)	$1427	$3091	–$1664	
		41	Async.	6 months - Calc.	DOT (clinic)	$1168	$1319	–$151	
				6 months - Calc.	DOT (field)	$1168	$3091	–$1923	
Nsengiyumva *et al*^19^ (2018)	Brazil	NR*	Sync.	6 months - DS	DOT (clinic)	$1107	$1815	–$709	S, D, T, F, I, O
				18 months - DR	DOT (clinic)	$21 817	$24 566	–$2748	
				9 months - TBI^G^	SAT	$695	$117	$578	
				9 months - TBI^C^	SAT	$681	$98	$584	
Nsengiyumva *et al*^20^ (2023)	Moldova	173	Async.	6 months - DS	DOT (clinic)	$865 ($489)	$956	–$91 (–$467)	S, T, F, I
		135	Async.	9 months - DR	DOT (clinic)	$1300 ($737)	$1434	–$134 (–$697)	
	Haiti	87	Async.	6 months - DS	DOT (clinic)	$2394 ($2251)	$859	$1535 ($1392)	
	Philippines	119	Async.	9 months - DR	DOT (clinic)	$1918 ($1436)	$49	$1868 ($1387)	
Siddiqui *et al*^36^ (2019)	USA	47	Async.	6 months - Calc.	DOT (field)	$4426	$5570	–$1145	S, T, F
Story *et al*^21^ (2019)	UK	50§	Sync.	6 months	DOT (clinic)	$2889	$10 010	–$7121	S, T, F
Wade *et al*^22^ (2012)	Australia	47§	Sync¶	5 months	DOT (field)	$2248	$2193	$55	S, T, F, O
						Societal costs per person treated			
Beeler Asay *et al*^34^ (2020)	USA	63	Async.	6 months	DOT (field)	$2707	$7053	–$4347	S, T, F, I, P
				6 months	DOT (clinic)	$2707	$3274	–$567	
		50	Sync.	6 months	DOT (field)	$1424	$7053	–$5629	
				6 months	DOT (clinic)	$1424	$3274	–$1849	
Nsengiyumva *et al*^19^ (2018)	Brazil	NR*	Sync.	6 months - DS	DOT (clinic)	$1402	$2438	–$1037	S, D, T, F, I, O, P
				18 months - DR	DOT (clinic)	$22 737	$26 645	–$3908	
				9 months - TBI^G^	SAT	$931	$326	$605	
				9 months - TBI^C^	SAT	$949	$353	$595	
Ravenscroft *et al*^24^ (2020)	Moldova	155	Async.	4 months	DOT (clinic)	$34	$128	–$94	P (transport only)
Salcedo *et al*^32^ (2021)	USA	100§	Async. (AI)	8 mo **	DOT (clinic and field)	$3139	$5759	–$2619	S, D, T, F, P

Costs included S, staff; D, drugs and treatment; T, travel and supplies; F, fixed assets and technology; I, implementation; O, overhead; P, patient expenses.

TBI C Cohort modelled based on persons who were close contacts of persons with contagious TB disease. G Cohort modelled as unselected persons with TBI.

Cost in parentheses are calculated with fixed costs annuitised over a 5-year useful lifespan.

* These patients were a modelled cohort.

† Patients given funds to cover cost of roundtrip to clinic on public transportation.

‡ This study observed costs from 28 patients but modelled a clinic assuming 15 patients per vehicle used in field DOT.

§ These studies observed costs from 112 patients (Story *et al*^21^), 58 patients (Wade *et al*^22^), 43 patients (Salcedo *et al*^32^) and modelled clinics with 50, 47 and 100 patients, respectively, as base cases. These studies also provided multiple scenarios with varying patients per clinic not shown here. 5X/week DOT is shown for Story *et al*.^21^

¶ Study considers 5% of patients remaining on DOT even in the DAT case.

** The model used observed treatment completion probabilities to model successful completion or required prolongation of treatment in each arm at the end of each month (minimum 5 months, maximum 16 months). Mean treatment durations in both groups were 8 months.

Async., asynchronous; Calc., converted from per observation cost (see Methods); DOT, directly observed therapy; DR, drug resistant; DS, drug susceptible; SAT, self-administered therapy; SMS, short message service; Sync., synchronous; TBI, TB infection.

Most VOT studies took place in high-income (12 of 16) or upper-middle-income (4 of 16) countries. Only one evaluated the use of VOT in a lower-middle-income country (LMIC).^20^ Nine studies evaluated synchronous VOT while eight assessed asynchronous VOT, with both yielding savings in most scenarios.

In the two studies that also considered costs from a societal perspective, the analyses suggested cost savings for treated persons and their families with VOT, leading to further overall savings. A third study evaluated the use of an asynchronous VOT technology that leveraged artificial intelligence (AI) software (branded AiCure) to screen the video recordings. From the societal perspective, VOT consistently yielded net savings over healthcare provider (field or clinic) DOT, but not compared with self-administered treatment. Two studies focused only on transportation costs reported by persons treated for TB; not surprisingly, VOT was then cheaper than clinic-based DOT^18 24^

A comparison of VOT and DOT costs per dose observation is shown in online supplemental table A5. Two VOT studies reported costs for both asynchronous and synchronous observation, compared in online supplemental table A6.

### Digital pillboxes

The provider costs for digital pillbox interventions are summarised in table 3. The range of provider costs was again extremely wide (ranging from international $118 to international $20 992 per person) but generally similar to the standard of care which usually included at least some component of SAT. The median incremental cost for digital pillbox use was a 20% increase over the standard of care. Unlike VOT, digital pillboxes were only reported from LMICs. While some studies evaluated digital pillboxes as a replacement for DOT, others considered it a means to augment DOT, for example, to support treatment of weekend doses which could not be directly observed. Only one study evaluated societal costs and suggested savings relative to clinic-based DOT (table 3). Another study that evaluated only patient expenses estimated that persons with TB who used a digital pillbox saved an average of $31 over the intensive phase of their treatment compared with the DOT group.^25^

**Table 3:** Provider and societal costs for digital adherence technologies (DATs) without video observation, and the standard of care (SoC) grouped by type of intervention in 2022 international dollars

Study	Country	DAT pts.	Cointervention	Duration	SOC	Costs per person treated			Costs included
						DAT	SoC	Incremental	
**Digital pillbox studies**	**Provider costs per person treated**		
Bahrainwala *et al*^40^ (2020)	Madagascar	475*	–	6 months	SAT	$4797	$4765	$32	S, D, T, F, I, O
		276*	DOT (clinic)+SAT	6 months	DOT (clinic)+SAT	$1914	$1862	$51	
Broomhead and Mars^38^ (2012)	S. Africa	24*	DOT (NR)	6 months	DOT alone (NR)	$1787	$2273	–$486	S, D, F, I
Mukora *et al*^49^ (2022)	S. Africa	1305†	–	6 months	SAT	$118	$67	$51	Abstract only
Nsengiyumva *et al*^19^ (2018)	Brazil	NR*	–	6 months - DS	DOT (clinic)	$826	$1815	–$990	S, D, T, F, I, O
				18 months - DR	DOT (clinic)	$20 992	$24 566	–$3574	
				9 mo - TBI^G^	SAT	$174	$98	$76	
				9 mo - TBI^C^	SAT	$187	$117	$70	
Saha *et al*^33^ (2022)	India	200	–	6 months	DOT (NR)	$370	$269	$101	S, T, F, I
Yang *et al*^39^ (2022)	Morocco	206	–	6 months	SAT	$1053	$411	$642	S, D, T, F, I, O
		NR‡	–	6 months	DOT+SAT§	$1968	$1635	$333	
						**Societal costs per person treated**			
Manyazewal *et al*^25^ (2022)	Ethiopia	52	–	2 months	DOT (clinic)	$1.85	$33	–$31	P
Nsengiyumva *et al*^19^ (2018)	Brazil	NR*	–	6 months - DS	DOT (clinic)	$1120	$2438	–$1318	S, D, T, F, I, O, P
				18 months - DR	DOT (clinic)	$21 911	$26 645	–$4734	
				9 months - TBI^G^	SAT	$422	$326	$96	
				9 months - TBI^C^	SAT	$441	$353	$88	
**SMS studies**						**Provider costs per person treated**			
Gashu *et al*^42^ (2021)	Ethiopia	131†	DOT (family)	4 months	N/A	$0.50			T (airtime only)
Louwagie *et al*^27^ (2022)	S. Africa	122	Motivational interviewing	3 months	SAT	$554	$119	$435	S, D, F, I, O
Nsengiyumva *et al*^19^ (2018)	Brazil	NR*	Patient reply	9 months - TBI^G^	SAT	$115	$98	$18	S, D, T, F, I, O
				9 months - TBI^C^	SAT	$127	$117	$10	
Peng *et al*^26^ 2014)	China	234	–	6 months	DOT (NR)	$71	$212	–$141	Abstract only
						**Societal costs per person treated**			
Nsengiyumva *et al*^19^ 2018)	Brazil	NR*	Patient reply	9 months - TBI^G^	SAT	$371	$326	$45	S, D, T, F, I, O, P
				9 months - TBI^C^	SAT	$386	$353	$33	
**99DOTS studies**						**Provider costs per person treated**			
Nsengiyumva *et al*^19^ (2018)	Brazil	NR*	–	6 months - DS	DOT (clinic)	$769	$1815	–$1046	S, D, T, F, I, O
				18 months - DR	DOT (clinic)	$20 916	$24 566	–$3650	
				9 months - TBI^G^	SAT	$117	$98	$20	
				9 months - TBI^C^	SAT	$131	$117	$14	
Nsengiyumva *et al*^20^ (2023)	Tanzania	976	–	6 months	DOT (family)	$469 ($439)	$0.00	$469 ($439)	S, T, F, I
	Bangladesh	719	–	6 months	DOT (clinic)	$280 ($231)	$211	$69 ($20)	
	Philippines	396	–	6 months	DOT (clinic)	$308 ($229)	$511	–$203 (–$281)	
Thompson *et al*^29^ (2022)	Uganda	1800/year*	–	6 months	DOT (family)	$963 ($163)	$0	$963 ($163)	S, T, F, I, O
Waswa *et al*^28^ (2022)	Uganda	1086	–	6 months - Calc.	None	$76			Abstract only¶
						**Societal costs per person treated**			
Nsengiyumva *et al*^19^ (2018)	Brazil	NR*	–	6 months - DS	DOT (clinic)	$672	$1539	–$868	S, D, T, F, I, O, P
				18 months - DR	DOT (clinic)	$13 787	$16 824	–$3037	
				9 months - TBI^G^	SAT	$230	$206	$25	
				9 months - TBI^C^	SAT	$243	$223	$20	
**Other studies**			**Intervention**			**Provider costs per person treated**			
Au-Yeung and DiCarlo^30^ (2012)	USA	NR*	Ingestible sensors	4 months	DOT (clinic)**	$1618	$2289	–$671	S, D, T, F
Daftary *et al*^31^ (2017)	Ethiopia	2300 (approx.)	Interactive voice calls	6 months - IPT	N/A	$148	–	–	T
						**Societal costs per person treated**			
Au–Yeung and DiCarlo^30^ (2012)	USA	NR*	Ingestible sensors	4 months	DOT (clinic)**	$1680	$3030	–$1350	S, D, T, F, P

Costs included S, staff; D, drugs and treatment; T, travel and supplies; F, fixed assets and technology; I, implementation; O, overhead; P, patient expenses).

TBI C Cohort modelled based on persons who were close contacts of persons with contagious TB disease. G Cohort modelled as unselected persons with TBI.

Cost in parentheses are calculated with fixed costs annuitised over a 5-year useful life.

* These patients were a modelled cohort. For Broomhead and Mars,^38^ while the 24 patients did receive the intervention in a pilot programme, the costs were entirely modelled using literature values. For Bahrainwala *et al*,^40^ the costs are presented per diagnosed patient only (to ensure comparability between other studies). Thompson *et al*^29^ used observed costs (from 891 intervention patients) and applied them to a modelled clinic with a service volume of 1800 patients per year.

† Target number of patients from protocol of parent randomised controlled trial.

‡ This study observed costs from an implementation of 206 patients but then modelled costs in a separate scenario considering costs of retreatment, MDR development and treatment, etc.

§ 2 months of DOT (facility details not reported) followed by 4 months of SAT. The model considers most patients as drug-sensitive (6-month treatment course); however, patients on retreatment are modelled to be treated for 8 months and MDR patients for 24 months.

¶ Costs described as ‘running costs only’ (ie, excluding start-up costs).

** 3X/week DOT is shown for Au-Yeung and DiCarlo.^30^

DR, drug-resistant; DS, drug susceptible; IPT, Isoniazid preventative therapy for TBI in people living with HIV; NR, not reported; SAT, self-administered therapy; SMS, short message service; TB, tuberculosis; TBI, TB infection.

### SMS-based interventions

The provider costs for SMS-based interventions are summarised in table 3. SMS was one of the lowest-cost interventions at a median cost of international $115 per person and was reported exclusively from LMICs. The standard of care comparator was mostly SAT, except in one study that compared SMS with DOT (type unspecified)^26^; this was the only study that found SMS cost saving. SMS-based interventions were used in several clinical contexts including TBI treatment (9 months of isoniazid), throughout treatment of drug-sensitive TB disease, or for continuation phase treatment only. In one study, the intervention consisted of motivational interviewing along with SMS reminders.^27^ The costs, therefore, include both the DAT and the interviewing cointervention. While most other studies involved one-way SMS reminders, one report modelled a two-way system where patients confirmed whether the dose was taken by replying to the reminder message.^19^ This study only evaluated the SMS intervention among persons treated for TBI. Only one study evaluated costs from a societal perspective (table 3) and found similar costs when compared with SAT.

### Medication sleeves with phone-based dose recording (branded 99DOTS)

Four studies analysed 99DOTS interventions in LMICs, and the costs are summarised in table 3. The provider costs for a 6-month regimen ranged from international $76 in Uganda when excluding start-up costs^28^ to $963 in another Uganda-based study that included those costs.^29^ The intervention was associated with cost savings in most cases when compared with clinic DOT as standard of care. In the reports by Thompson *et al* and Nsengiyumva *et al* (2023; Tanzania group), there were no provider costs for DOT since the treatment observers were unpaid family members. The two studies also considered scenarios where fixed costs were annuitised over 5 years. Nsengiyumva *et al* (2023) also considered scenarios for scale-up using larger theoretical patient numbers that are not presented here. Only one study^19^ considered societal costs; it suggested additional savings for persons treated for TB disease.

### Other technologies (interactive voice calls and ingestible sensors)

One modelling study evaluated total treatment costs using a hypothetical target cost for ingestible sensors to detect and promote adherence to TB disease treatment.^30^ This study modelled a clinic in a high-income country (USA) using ingestible sensors compared with clinic DOT as the standard of care. Another study provided feature phones and 6 months of airtime to support an interactive voice response intervention to promote adherence to TBI treatment.^31^ The provider costs from these studies are highlighted in table 3 along with results from the societal perspective reported by the ingestible sensors study.

A visual summary of all incremental costs to providers is illustrated in online supplemental figures A1 and A2. Some patterns become more evident visually. VOT was most often compared with some form of DOT, while other DATs were often compared with SAT. Not surprisingly, health provider savings with VOT were more pronounced when it was compared with field DOT, as opposed to facility-based DOT.

### Cost-effectiveness

Among the nine studies that addressed cost-effectiveness, outcomes assessed varied considerably. Four modelling studies estimated a cost per DALY averted; results are summarised in online supplemental table A7. Two modelling studies also estimated cost per TB case averted, largely among persons treated for TBI (online supplemental table A8)

Three studies considered cost per QALY gained (online supplemental table A9). One of these was a trial which found no significant effect of an SMS intervention plus motivational interviewing on health utility scores.^27^ Hence the authors did not estimate cost per QALY gained; for completeness, we have done so using the point estimates they provided (online supplemental table A9). In a probabilistic sensitivity analysis, another study found that an AI-based VOT application^32^ was dominant in 93.5% of simulations, that is, cheaper with better health outcomes, compared with provider (clinic and field) DOT. In India, digital pillboxes were judged to be highly cost-effective compared with DOT.^33^

One study compared synchronous VOT to field DOT and estimated it would cost international $1.12 per additional treatment observation accomplished (95% CI = $0.43 to $1.91) to implement VOT in a clinic of 47 patients.^22^

Another study compared 99DOTS to family DOT in Uganda and estimated it would cost $1128 per additional treatment success (95% CI = $697 to $1982) to implement the intervention in a clinic over 5 years.^29^ Importantly, however, this study used the per-protocol effect of this intervention, since the intention-to-treat analysis did not show improved treatment success with the DAT intervention in the parent randomised controlled trial.

### Subgroups and special populations

Few studies included persons treated for TBI. Several combined data for persons treated for TB disease and TBI, to estimate costs per observed treatment dose for their entire cohort^34^,^36^ or presented estimates for a typical drug-sensitive treatment course.^37^ One study reported on a group of persons living with HIV who received isoniazid preventive therapy for TBI.^31^ Only one study modelled costs and cost-effectiveness separately for persons treated for TBI and found all DATs studied to be less cost-effective for TBI compared with active TB disease.^19^ Of note, the standard of care was SAT for TBI and clinic-based DOT for active TB disease. This was also one of two studies that analysed costs or cost-effectiveness separately for persons with drug-resistant TB disease; both studies suggested greater savings when DATs replaced clinic-based DOT for treatment support for drug-resistant TB, as compared with drug-sensitive disease.^19 20^ Two studies modelled the development of acquired drug resistance among persons treated for TB disease and included the ensuing treatment costs.^38 39^ Several other studies combined data for persons with drug-sensitive and drug-resistant TB disease, with the latter group representing less than 5% of each cohort.^24 34 37^

Only three studies included persons under the age of 16^20 36 40^ and none performed any subgroup analysis according to age. As mentioned above, one study focused exclusively on isoniazid preventive therapy among persons living with HIV.^31^ Two other cohorts included >10% of persons living with HIV but did not report any separate data for this subgroup.^25 27^ Only two studies reported including any persons with extrapulmonary TB disease but again did not report data specific to this subgroup.^21 24^

### International dollar versus USD estimates

In LMIC settings, as expected the cost estimates in USD using market exchange rates (online supplemental tables A10–A21) were 39%–74% lower than those expressed in international dollars, using PPP. The difference was most marked for the 99DOTS medication sleeve intervention, which was exclusively used in lower-income and middle-income settings and where the median provider cost of the intervention was 73% lower in USD. The median provider costs of digital pillbox and SMS interventions were 63% and 50% lower, respectively. The cost-effectiveness estimates for these three interventions improved accordingly. As VOT cost estimates came from high-income countries, and those for the other DATs from LMIC settings, the use of market exchange rates magnified cost differences between VOT and the lower-cost technologies.

### Quality of reporting

The results of the assessment for quality of reporting, using the CHEERS 2022 checklist,^17^ are summarised in online supplemental table A22. The proportion of checklist items addressed ranged from 30% to 89% (median 68%). Five studies reported conflicts of interest, where a listed author was either an inventor of the DAT evaluated, or an employee of a company that manufactured it.^22 30 33 37 41^ Two studies did not report on conflicts of interest.^23 36^

## Discussion

Of the technologies currently used by TB programmes, VOT was reported as cost saving relative to clinic or field DOT in most studies, particularly with respect to cost per dose observed. However, VOT was primarily evaluated in high-income or upper-middle-income countries with well-established DOT programmes, and technical infrastructure that can support VOT. In these locations, VOT can potentially be considered a more efficient, lower-cost option for monitoring treatment, particularly in rural and remote areas.

It is unclear whether these results are generalisable to lower-income settings where labour savings relative to in-person DOT may not offset equipment and infrastructure costs. However, VOT or other DATs requiring smartphone access could be adapted to reduce some infrastructure costs. For example, if local privacy laws allow, patients could use one of many free encrypted video call applications as opposed to paid applications. Patients could opt for video calls or video messages if they feel comfortable doing so and already have access to mobile internet. Low-cost or used smartphones could also be loaned to patients who do not already have one. Of course, from the provider perspective any treatment support provided by the health system, whether with a DAT or DOT, is more expensive than SAT.

In theory, costs for asynchronous VOT could differ from those for synchronous observation. Asynchronous VOT requires dedicated software and video storage but could allow further labour efficiencies if videos are watched consecutively or are screened by AI software. In fact, both asynchronous and synchronous VOT were reported to be cost saving in higher-income settings. Indeed, the two studies that analysed both asynchronous and synchronous VOT generally found very similar costs per dose observed.

Digital pillbox costs were studied exclusively in LMICs, where they were occasionally used to supplement DOT. They were often judged cost saving when compared with DOT, particularly when costs to persons with TB were considered. On the other hand, they were not cost-effective or cost-saving when compared with SAT. Similarly, perhaps reflecting their low cost, SMS intervention costs were reported exclusively from LMICs, and most often compared with SAT. However, interpretation was limited by significant cointerventions^27^ or by restricted types of data available.^26 42^ 99DOTS medication sleeves were also used primarily in LMICs, with estimated costs that were generally similar to or lower than those for clinic-based DOT. Hence digital pillboxes, SMS interventions and 99DOTS may be affordable or even cost saving in lower-income settings. However, their impact on health outcomes will also need to be considered.

The incremental cost of treatment support with DATs varied dramatically with the standard of care comparator and the setting. For example, savings to the healthcare system were typically greatest when DATs replaced field DOT (where the burden of travel is on the provider), as opposed to clinic DOT. There were also far more studies comparing DATs (particularly VOT) to DOT than to SAT. Each DAT was most often deemed cost saving for providers when compared with clinic or field DOT, reflecting potential reductions in labour and travel costs. Given the considerable challenges in providing in-person DOT in LMICs,^43^,^45^ where most people with TB live, it is important to understand how the costs of the DATs compare to those for both DOT and SAT in the same settings. However, for local health departments already using DOT or considering implementing it, DATs could offer less intrusive, more efficient alternatives.

Other important sources of heterogeneity between studies included the number of persons served by a given programme or intervention, and lifespan attributed to equipment. For example, some studies considered only the persons served during a short trial period while others considered larger-scale operations at multiple clinics over longer time frames, including sensitivity analyses that evaluated different levels of scale-up.^20^,^22^ Some studies noted that costs per person served fell dramatically if fixed equipment continued to be used beyond initial study timelines.^20 29^

The use of international dollars as opposed to USD from market exchange rates (reported in online supplemental section S5) resulted in higher DAT and comparator costs for studies conducted in LMICs. This is because PPP corrects for differences in resource prices between settings. Hence median costs for digital pillboxes, 99DOTS and SMS were 2–4 times higher in international dollars than in USD based on market exchange rates. The resulting cost estimates were, therefore, closer to (though still consistently lower than) those for VOT which was mostly used in the USA. Cost-effectiveness estimates from LMICs showed a similar pattern.

Overall, the quality of reporting was limited when studies were assessed against the CHEERS checklist—a standard for health economic evaluations. For example, nearly half the studies we found did not report a currency year. Others incorrectly reported the perspective of their analyses, did not fully describe key data inputs, did not include relevant cost components or contained apparent calculation errors.

### Strengths and limitations

To our knowledge, this is the first systematic review focusing specifically on cost and cost-effectiveness estimates for the application of DATs to TB treatment. We used a wide-ranging search strategy across multiple databases, without language restriction and with inclusion of suitable publications from the grey literature—although the inclusion of conference abstracts limited the scope of information and quality assessment for those specific reports. Every step of our review, from filtering of titles and abstracts through data extraction, reporting and quality assessment, involved two independent reviewers, with disagreements resolved by consensus, and a third senior reviewer when needed.

To support comparisons between study findings, we expressed all costs in the same currencies (2022 international dollars as well as 2022 USD). Similarly, we explicitly tabulated which cost components, such as technology and equipment, staff time, overheads and patient/family costs were included in each study. Whenever possible, we summarised the cost of both the DAT and the comparator used for treatment support in each study. We also used a standard and widely used health economic analysis checklist to assess the quality of the reports included.

Because of the marked diversity of interventions, comparators, study settings, study designs and cost measures, it was neither possible nor appropriate to pool study results through meta-analysis. To enhance comparability of results across studies, we focused on incremental (as opposed to absolute) costs, and on costs per person (as opposed to cost per dose observation or aggregate costs across all persons treated). Inevitably, there remain differences in methods and assumptions between individual studies. For example, covering the cost of a smartphone purchase makes a DAT intervention more expensive, compared with only including persons who own a smartphone. Similarly, the longer the assumed lifespan for DAT hardware, the lower the cost per person.

For the cost-effectiveness analyses, in particular, robust effectiveness data were often lacking. These analyses often involved the modelling of downstream costs and health outcomes, with many underlying (and unproven) assumptions. This was reflected in the diversity of results from these studies. Finally, there are likely other grey literature reports that were missed by our search.

## Conclusions

Cost and cost-effectiveness analyses for the DATs currently used by TB programs have yielded variable results, particularly when compared with conventional directly observed treatment. Studies have often involved small numbers of affected persons or specialised settings, hampering their generalisability. VOT was more consistently associated with cost savings compared with clinic or field DOT in higher-income settings, related to reductions in travel and labour expenses for healthcare workers, and in productivity losses for persons on treatment. However, few analyses have considered costs borne by affected individuals and their families, so the overall potential for societal cost savings has not been adequately characterised. Any such savings are only relevant to the extent that treatment supported by DATs is associated with similar or better outcomes than the existing local standard of care. Moreover, any attendant health gains or cost savings can only be realised if the necessary technical and human resources are in place and if barriers to DAT uptake are mitigated. It is also clear that more and higher-quality operations research focusing on costs is needed, particularly in communities and settings that carry the greatest burden of TB.

### Changes to protocol

A sensitivity analysis with and without conference abstracts was not performed since only three abstracts were included and no meta-analysis was performed. Abstracts were instead clearly labelled in the results tables. A sensitivity analysis with market-based exchange rates was added. Certainty of evidence was not rated formally using criteria of the Grading of Recommendations, Assessment, Development and Evaluation approach since results were not pooled and cost-effectiveness outcomes were mostly not comparable.

## Supplementary material

10.1136/bmjgh-2024-015654 online supplemental file 1

## Data Availability

All data relevant to the study are included in the article or uploaded as online supplemental information.
