# Supplementary material for: Cost and cost-effectiveness of digital technologies for support of tuberculosis treatment adherence: a systematic review
Source: BMJ Glob Health. 2024 Oct 30;9(10):e015654. doi: 10.1136/bmjgh-2024-015654 (PMC11529515; doi:10.1136/bmjgh-2024-015654)
Supplement: online supplemental file 1 [file bmjgh-9-10-s001.pdf]

Cost and Cost-Effectiveness of Digital Technologies for Support of Tuberculosis Treatment Adherence: A Systematic Review

Supplementary Materials

Cedric Kafie [1], Mona Salaheldin Mohamed[1], Miranda Zary[1], Chimweta Ian Chilala [2], Shruti Bahukudumbi[3], Genevieve Gore[4], Nicola Foster[2], Katherine Fielding [2], Ramnath Subbaraman[3,5], and Kevin Schwartzman[1]

[1]McGill International Tuberculosis Centre; Research Institute of the McGill University Health Centre, Montréal, Canada
[2]TB Centre, London School of Hygiene and Tropical Medicine, London, UK
[3]Department of Public Health and Community Medicine, Tufts University School of Medicine, Boston, USA
[4]Schulich Library of Physical Sciences, Life Sciences, and Engineering; McGill University, Montréal, Canada
[5]Division of Geographic Medicine and Infectious Diseases, Tufts Medical Center, Boston, USA

Data Supplement 1: Supplementary material

S1 Search strategy

MEDLINE (Ovid)

Ovid MEDLINE(R) ALL <1946 to April 18, 2023>

1 exp tuberculosis/ or mycobacterium tuberculosis/ 228099

2 Antitubercular Agents/ 41251

3 (Tuberculosis or Kochs disease or Phthisis or TB or MTB or MDRTB or XDRTB or DRTB or LTBI or directly observed treatment short course).mp. 300916

4 1 or 2 or 3 [TB CONCEPT] 306343

5 ((antitubercular agents/ or directly observed therapy/ or medication adherence/ or patient compliance/ or "treatment adherence and compliance"/) and technology/) or mobile applications/ or internet/ or cell phone/ or smartphone/ or text messaging/ or computer, handheld/ or telemedicine/ or therapy, computer-assisted/ or medical informatics applications/ 148201

6 (((digital* or electronic* or mobile or wireless* or virtual*) adj2 (adherence or medication monitor* or medication package* or observ*)) or digital technolog* or technology-based or Digital health or eHealth or e health or mhealth or m health or SMS or reminder* or short messag* service* or text messag* or MMS or multimedia messag* or MEMS or (monitor* adj2 (electronic* or sensor* or device*)) or Webcam* or web cam* or smartphone* or smart phone* or web based or health IT or health ICT or ((cell* or mobile) adj1 (device* or health or phone* or technolog*)) or video* or cellphone* or feature phone* or vdot or vmalt or tele* or dat or wot or 99dots or 99 dots or monitoring system* or ingestible sensor* or merm or artificial intelligence or ai or vot or ((digital or smart) adj (pill box* or pillbox*)) or VST).mp. 705421

7 5 or 6 [DAT CONCEPT] 772859

8 4 and 7 [TB CONCEPT AND DAT CONCEPT] 3219

9 limit 8 to yr="2000 -Current" 2791

10 Economics/ or exp "Costs and Cost Analysis"/ or Economics, Nursing/ or Economics, Medical/ or Economics, Pharmaceutical/ or exp Economics, Hospital/ or Economics, Dental/ or exp "Fees and Charges"/ or exp Budgets/ or exp models, economic/ or markov chains/ or monte carlo method/ or exp Decision Theory/ 394969

11 (Cost* or health payer* or societal or Economic* or Dominant or Dominated or Increment* or additional health gain* or Expense* or Budget* or Financi* or Benefit* or QALY*).mp. 2703781

12 (health utilit* or life year* or markov or monte carlo or pharmacoeconomic* or expenditure* or finance* or price or prices or pricing or (value adj2 (money or monetary)) or (decision* adj2 (tree* or analy* or model*))).mp.301385

13 or/10-12 [COST OR COST EFFECTIVENESS CONCEPT] 2875832

14 9 and 13 [TB CONCEPT AND DAT CONCEPT AND COST OR COST EFFECTIVENESS CONCEPT] 541

15 ("20220414" or "20220415" or "20220416" or "20220417" or "20220418" or "20220419" or 2022042* or 2022043* or 202205* or 202206* or 202207* or 202208* or 202209* or 20221* or 2023*).dt,ez,da. 1902583

16 14 and 15 82

https://ovidsp.ovid.com/ovidweb.cgi?T=JS&NEWS=N&PAGE=main&SHAREDSEARCHID=6Q0B8MrdSrEDSTHM9bRCUAwt58PjIrf3BXkSsDkuyGVifkVpHoqsrdglq7yGyHOJd

Embase (Ovid)

Embase <1996 to 2023 Week 15>

1 exp tuberculosis/ or mycobacterium tuberculosis/ 204337

2 tuberculostatic agent/ 30987

3 (Tuberculosis or Kochs disease or Phthisis or TB or MTB or MDRTB or XDRTB or DRTB or LTBI or directly observed treatment short course).mp. 246187

4 1 or 2 or 3 [TB CONCEPT] 257637

5 ((tuberculostatic agent/ or directly observed therapy/ or medication compliance/ or patient compliance/) and technology/) or communication technology/ or exp mobile application/ or internet/ or web-based intervention/ or exp mobile phone/ or text messaging/ or personal digital assistant/ or telemedicine/ or exp teleconsultation/ or telemonitoring/ or video consultation/ or computer-assisted therapy/ or computer-assisted drug therapy/ or medical informatics/ 261570

6 (((digital* or electronic* or mobile or wireless* or virtual*) adj2 (adherence or medication monitor* or medication package* or observ*)) or digital technolog* or technology-based or Digital health or eHealth or e health or mhealth or m health or SMS or reminder* or short messag* service* or text messag* or MMS or multimedia messag* or MEMS or (monitor* adj2 (electronic* or sensor* or device*)) or Webcam* or web cam* or smartphone* or smart phone* or web based or health IT or health ICT or ((cell* or mobile) adj1 (device* or health or phone* or technolog*)) or video* or cellphone* or feature phone* or vdot or vmalt or tele* or dat or wot or 99dots or 99 dots or monitoring system* or ingestible sensor* or merm or artificial intelligence or ai or vot or ((digital or smart) adj (pill box* or pillbox*)) or VST).mp. 870210

7 5 or 6 [DAT CONCEPT] 984663

8 4 and 7 [TB CONCEPT AND DAT CONCEPT] 5167

9 limit 8 to yr="2000 -Current" 5020

10 Economics/ or exp economic evaluation/ or exp *health economics/ or budget/ or exp economic model/ or exp markov chain/ or exp monte carlo method/ or Decision Theory/ 654528

11 (Cost* or health payer* or societal or Economic* or Dominant or Dominated or Increment* or additional health gain* or Expense* or Budget* or Financi* or Benefit* or QALY*).ti,ab,kf. 2903252

12 (health utilit* or life year* or markov or monte carlo or pharmacoeconomic* or expenditure* or finance* or price or prices or pricing or (value adj2 (money or monetary)) or (decision* adj2 (tree* or analy* or model*))).mp.434885

13 10 or 11 or 12 3379669

14 9 and 13 933

15 limit 14 to dc=20220414-20230419 131

https://ovidsp.ovid.com/ovidweb.cgi?T=JS&NEWS=N&PAGE=main&SHAREDSEARCHID=3DgeO1F774ECCcRfcqeXXAzvP09LOq5uzvf6SO0bpEKgOafK0X9ehQXyW219thL2h

CINAHL (EBSCOhost)

#	Query	Limiters/Expanders	Last Run Via	Results
S16	S14 AND S15	Expanders - Apply equivalent subjects Search modes - Boolean/Phrase	Interface - EBSCOhost Research Databases Search Screen - Advanced Search Database - CINAHL Plus with Full Text	39
S15	EM 20220414- OR ZD "in process"	Expanders - Apply equivalent subjects Search modes - Boolean/Phrase	Interface - EBSCOhost Research Databases Search Screen - Advanced Search Database - CINAHL Plus with Full Text	1,147,669
S14	S9 AND S13	Expanders - Apply equivalent subjects Search modes - Boolean/Phrase	Interface - EBSCOhost Research Databases Search Screen - Advanced Search Database - CINAHL Plus with Full Text	179
S13	S10 OR S11 OR S12	Expanders - Apply equivalent subjects Search modes - Boolean/Phrase	Interface - EBSCOhost Research Databases Search Screen - Advanced Search Database - CINAHL Plus with Full Text	Display
S12	("health utilit*" OR "life year*" OR markov OR "monte carlo" OR pharmacoeconomic* OR expenditure* OR finance* OR price OR prices OR pricing OR (value N2 (money OR monetary)) OR (decision* N2 (tree* OR analy* OR model*)))	Expanders - Apply equivalent subjects Search modes - Boolean/Phrase	Interface - EBSCOhost Research Databases Search Screen - Advanced Search Database - CINAHL Plus with Full Text	Display
S11	(Cost* OR "health payer*" OR societal OR Economic* OR Dominant OR Dominated OR Increment* OR "additional health gain*" OR Expense* OR Budget* OR Financi* OR Benefit* OR QALY*)	Expanders - Apply equivalent subjects Search modes - Boolean/Phrase	Interface - EBSCOhost Research Databases Search Screen - Advanced Search Database - CINAHL Plus with Full Text	Display
S10	(MH "Economics") OR (MH "Costs and Cost Analysis+") OR (MH "Economic Aspects of Illness") OR (MH "Fees and Charges+") OR (MH "Budgets") OR (MH "Decision Trees+")	Expanders - Apply equivalent subjects Search modes - Boolean/Phrase	Interface - EBSCOhost Research Databases Search Screen - Advanced Search Database - CINAHL Plus with Full Text	Display
S9	S4 AND S7	Limiters - Published Date: 20000101-20231231 Expanders -	Interface - EBSCOhost Research Databases Search Screen - Advanced Search	858

		Apply equivalent subjects<br>Search modes - Boolean/Phrase	Database - CINAHL Plus with Full Text	
S8	S4 AND S7	Expanders - Apply equivalent subjects<br>Search modes - Boolean/Phrase	Interface - EBSCOhost Research Databases<br>Search Screen - Advanced Search<br>Database - CINAHL Plus with Full Text	Display
S7	S5 OR S6	Expanders - Apply equivalent subjects<br>Search modes - Boolean/Phrase	Interface - EBSCOhost Research Databases<br>Search Screen - Advanced Search<br>Database - CINAHL Plus with Full Text	Display
S6	(((digital* OR electronic* OR mobile OR wireless* OR virtual*) N2 (adherence OR "medication monitor*" OR "medication package*" OR observ*)) OR "digital technolog*" OR technology-based OR "Digital health" OR eHealth OR "e health" OR mhealth OR "m health" OR SMS OR reminder* OR "short messag* service*" OR "text messag*" OR MMS OR "multimedia messag*" OR MEMS OR (monitor* N2 (electronic* OR sensor* OR device*)) OR Webcam* OR "web cam*" OR smartphone* OR "smart phone*" OR "web based" OR "health IT" OR "health ICT" OR ((cell* OR mobile) N1 (device* OR health OR phone* OR technolog*)) OR video* OR cellphone* OR "feature phone*" OR vdot OR vmalt OR tele* OR dat OR wot OR 99dots OR "99 dots" OR "monitoring system*" OR "ingestible sensor*" OR merm OR "artificial intelligence" OR ai OR vot OR ((digital OR smart) W1 ("pill box*" OR pillbox*)) OR VST)	Expanders - Apply equivalent subjects<br>Search modes - Boolean/Phrase	Interface - EBSCOhost Research Databases<br>Search Screen - Advanced Search<br>Database - CINAHL Plus with Full Text	Display
S5	((MH "Antitubercular Agents") OR (MH "Directly Observed Therapy") OR (MH "Medication Compliance") OR (MH "Patient Compliance")) AND (MH "Technology")) OR (MH "Wireless Communications") OR (MH "Mobile Applications") OR (MH "Internet") OR (MH "Internet-Based Intervention") OR (MH "Cellular Phone+") OR (MH "Text Messaging+") OR (MH "Instant Messaging") OR (MH "Interactive Voice Response Systems") OR (MH "Videoconferencing+") OR (MH "Computers, Hand-Held+") OR (MH "Telehealth+") OR (MH "Digital Technology") OR (MH "Therapy, Computer Assisted") OR (MH "Drug Therapy, Computer Assisted") OR (MH "Medical Informatics")	Expanders - Apply equivalent subjects<br>Search modes - Boolean/Phrase	Interface - EBSCOhost Research Databases<br>Search Screen - Advanced Search<br>Database - CINAHL Plus with Full Text	Display
S4	S1 OR S2 OR S3	Expanders - Apply equivalent subjects	Interface - EBSCOhost Research Databases<br>Search Screen - Advanced Search	Display

		Search modes - Boolean/Phrase	Database - CINAHL Plus with Full Text	
S3	(Tuberculosis OR "Kochs disease" OR Phthisis OR TB OR MTB OR MDRTB OR XDRTB OR DRTB OR LTBI OR "directly observed treatment short course")	Expanders - Apply equivalent subjects<br>Search modes - Boolean/Phrase	Interface - EBSCOhost Research Databases<br>Search Screen - Advanced Search<br>Database - CINAHL Plus with Full Text	Display
S2	(MH "Antitubercular Agents")	Expanders - Apply equivalent subjects<br>Search modes - Boolean/Phrase	Interface - EBSCOhost Research Databases<br>Search Screen - Advanced Search<br>Database - CINAHL Plus with Full Text	Display
S1	(MH "Tuberculosis+") OR (MH "Mycobacterium Tuberculosis")	Expanders - Apply equivalent subjects<br>Search modes - Boolean/Phrase	Interface - EBSCOhost Research Databases<br>Search Screen - Advanced Search<br>Database - CINAHL Plus with Full Text	Display

CENTRAL (Cochrane Library/Wiley)

Search Name:

Date Run: 19/04/2023 19:42:11

Comment:

ID Search Hits

#1 (Tuberculosis or "Kochs disease" or Phthisis or TB or MTB or MDRTB or XDRTB or DRTB or LTBI or "directly observed treatment short course"):ti,ab,kw 9141

#2 (((digital* or electronic* or mobile or wireless* or virtual*) NEAR/2 (adherence or medication NEXT monitor* or medication NEXT package* or observ*)) or digital NEXT technolog* or technology-based or "Digital health" or eHealth or "e health" or mhealth or "m health" or SMS or reminder* or short NEXT messag* NEXT service* or text NEXT messag* or MMS or multimedia NEXT messag* or MEMS or (monitor* NEAR/2 (electronic* or sensor* or device*)) or Webcam* or web NEXT cam* or smartphone* or smart NEXT phone* or "web based" or "health IT" or "health ICT" or ((cell* or mobile) NEXT (device* or health or phone* or technolog*)) or video* or cellphone* or feature NEXT phone* or vdot or vmalt or tele* or dat or wot or 99dots or 99 NEXT dots or monitoring NEXT system* or ingestible NEXT sensor* or merm or artificial NEXT intelligence or ai or vot or ((digital or smart) NEXT (pill box* or pillbox*)) or VST):ti,ab,kw 359210

#3 #1 AND #2 with Publication Year from 2000 to 2023, in Trials 2118

#4 (Cost* or health NEXT payer* or societal or Economic* or Dominant or Dominated or Increment* or additional NEXT health NEXT gain* or Expense* or Budget* or Financi* or Benefit* or QALY*) 268651

#5 (health NEXT utilit* or life NEXT year* or markov or "monte carlo" or pharmacoeconomic* or expenditure* or finance* or price or prices or pricing or (value NEAR/2 (money or monetary)) or (decision* NEAR/2 (tree* or analy* or model*))):ti,ab,kw 23914

#6 #4 OR #5 277520

#7 #3 AND #6 639

Date added to CENTRAL trials database

Custom Range: 14/04/2022 to 19/04/2023

55 records

WOS.SCI,WOS.ISTP,WOS.ESCI (Web of Science Core Collection)

Web of Science Search Strategy (v0.1)

Search: TS=(Tuberculosis OR "Kochs disease" OR Phthisis OR TB OR MTB OR MDRTB OR XDRTB OR DRTB OR LTBI OR "directly observed treatment short course")

AND

TS=(((digital* OR electronic* OR mobile OR wireless* OR virtual*) NEAR/2 (adherence OR "medication monitor*" OR "medication package*" OR observ*)) OR "digital technolog*" OR technology-based OR "Digital health" OR eHealth OR "e health" OR mhealth OR "m health" OR SMS OR reminder* OR "short messag* service*" OR "text messag*" OR MMS OR "multimedia messag*" OR MEMS OR (monitor* NEAR/2 (electronic* OR sensor* OR device*)) OR Webcam* OR "web cam*" OR smartphone* OR "smart phone*" OR "web based" OR "health IT" OR "health ICT" OR ((cell* OR mobile) NEAR/1 (device* OR health OR phone* OR technolog*)) OR video* OR cellphone* OR "feature phone*" OR vdot OR vmalt OR tele* OR dat OR wot OR 99dots OR "99 dots" OR "monitoring system*" OR "ingestible sensor*" OR merm OR "artificial intelligence" OR ai OR vot OR ((digital OR smart) NEAR/0 ("pill box*" OR pillbox*)) OR VST)

AND

(

TS=(Cost* OR "health payer*" OR societal OR Economic* OR Dominant OR Dominated OR Increment* OR "additional health gain*" OR Expense* OR Budget* OR Financi* OR Benefit* OR QALY*)

OR

TS=("health utilit*" OR "life year*" OR markov OR "monte carlo" OR pharmacoeconomic* OR expenditure* OR finance* OR price OR prices OR pricing OR (value N2 (money OR monetary)) OR (decision* N2 (tree* OR analy* OR model*)))

)

Editions: WOS.SCI,WOS.ISTP,WOS.ESCI

Timespan: 2000-01-01 to 2023-04-19

Date Run: Thu Apr 20 2023 16:05:49 GMT-0400 (Eastern Daylight Time)

Results: 563

Database: Web of Science Core Collection

Entitlements:

- WOS.IC: 1993 to 2023

- WOS.CCR: 1985 to 2023

- WOS.SCI: 1900 to 2023

- WOS.AHCI: 1975 to 2023

- WOS.BHCI: 2005 to 2023

- WOS.BSCI: 2005 to 2023

- WOS.ESCI: 2005 to 2023

- WOS.ISTP: 1990 to 2023

- WOS.SSCI: 1900 to 2023

- WOS.ISSHP: 1990 to 2023

Searches:

Search:

#1

Timespan: 2022-04-14 to 2023-04-19

Date Run: Thu Apr 20 2023 16:07:32 GMT-0400 (Eastern Daylight Time)

Results: 70

MedRxiv and other preprints via Europe PMC

(TITLE:Tuberculosis OR TITLE:"Kochs disease" OR TITLE:Phthisis OR TITLE:TB OR TITLE:MTB OR TITLE:MDRTB OR TITLE:XDRTB OR TITLE:DRTB OR TITLE:LTBI OR TITLE:"directly observed treatment short course" OR TITLE:"antituberculosis" OR TITLE:"antituberculous") AND (Adher* OR "directly observed" OR Digital OR electronic OR internet OR mobile OR wireless OR virtual OR TITLE:technology OR "technology based" OR tele* OR ehealth OR "e health" OR mhealth OR "m health" OR SMS OR reminder* OR messaging OR message* OR MEMS OR web OR webcam* OR smartphone* OR "health IT" OR "health ICT" OR video* OR cellphone* OR phone* OR vdot OR vmalt OR dat OR wot OR 99dots OR "99 dots" OR "monitoring system" OR "monitoring systems" OR "ingestible sensor" OR "ingestible

sensors" OR merm OR "artificial intelligence" OR AI OR VOT OR "smart pillbox" OR "smart pill box" OR VST OR "computer assisted") AND (SRC:PPR) AND CREATION_DATE:[2022-04-19 TO 2023-04-19]

Clinicaltrials.gov basic search

Translated and exported to EndNote/RIS and documented

384 records on April 25, 2023

384 Studies found for: **Adherence OR directly observed OR computer OR Digital OR electronic OR internet OR mobile OR virtual OR technology OR video OR mhealth OR artificial intelligence OR AI OR cellphones OR SMS OR reminders OR monitoring OR MEMS OR DAT OR sensors | Tuberculosis OR Kochs disease OR Phthisis OR TB OR MTB OR MDRTB OR XDRTB OR DRTB OR LTBI OR directly observed treatment short course**

S2 Definition for inclusion of digital adherence technologies

Intervention can be patient-facing only, provider-facing only, or patient and provider-facing.

Intervention includes:

- a digital component (which could be part of a multi-component intervention) that goes beyond a means of non-automated voice or text communication between a human operator and a patient (i.e. more than a manual phone call, text message or email)
- with the intention to promote treatment adherence and/or reducing missed visits and/or reducing LTFU (and thereby improving successful treatment outcomes)

Examples of "digital adherence component" included (though not limited to)

- SMS daily reminders (1- or 2-way) to patient to take treatment
- SMS reminders (1- or 2-way) to patient to attend dispensing visit/routine follow-up appointment
- Automated phone calls to remind patient for visit/daily dose
- Smart pillbox daily reminders to patient to take treatment
- Smart pillbox reminders to patient attend dispensing visit/routine follow-up appointment
- Chatbot/telemedicine accessed by TB patients that provides information about "treatment adherence"
- Automated feedback (such as electronic health record) to alert HCW to a missed visit (eg., dispensing, routine follow-up appt for patients on TB/LTBI treatment) by a patient with an intended action of "promoting treatment adherence and/or reducing LTFU"
- Digital calendar generated (from electronic health record/smart pill box) showing missed visits/doses by patient at consultation with HCW
- Telehealth – video-call initiated by HCW
- WhatsApp group (with patients/HCWs)- educational messages sent etc..
- Specialist Healthcare provider Apps (– not automated) – used by patient and HCW

These need to be used *in conjunction* with the intention of promoting treatment adherence and/or reducing missed visits and/or reducing LTFU (and thereby improving successful treatment outcomes)

Examples of excluded interventions (not limited to)

- Electronic health record to document (monitor/record/summarise) visit attendance only - with NO intended action of “promote treatment adherence and/or reducing LTFU”
- Mobile technology to collect data (TB register etc..) with no feedback loop to promote adherence
- Non-automated “routine telephone calls” to patient
- Phone calls or SMS or WhatsApp messages that are not automated (i.e. written and sent by a human) - with no other digital component

S3 Cost component checklist

Table A1: Cost component checklist

**Category**	**Item**
Staff	Healthcare worker salary & benefits
TB treatment	TB treatment costs (drugs, follow-up lab tests)
Travel and Supplies	DAT-specific supplies (if applicable, e.g. phone number medication sleeves)
	Technology supplied to patients that is not recouped (e.g. given a phone, tablet, wireless sensor)
	Travel costs (during operation, if applicable)
	Airtime, data, and other variable fees
Fixed assets & technology	DAT technology support (& software-as-a-service fees)
	DAT components (e.g. computers, projectors, smartphones, software, items in the clinic)
	DAT technology servers (if applicable)
Design & Implementation	DAT design and production
	Training costs - salaries of health workers and the trainers, lodging, travel, training materials
	Travel costs (during implementation)
Overhead	Rent (or building depreciation)
	Utilities
	General administrative supplies
Other provider costs	Additional costs not captured above - Health Care System
Out-of-pocket patient costs	Out-of-pocket healthcare costs (drugs, fees)
	Travel costs
	Data & airtime expenses
	Technology purchased by patients
Indirect patient costs	Lost productivity (e.g. time off work)
Other patient costs	Additional costs not captured above - Patients / Family

Table A2: Cost multipliers used to express estimates in common units

**Authors**	**Year**	**Country**	**Currency & Year Reported**	**Local currency**	**A. Exchange rate multiplier for local currency**	**B. Local Inflation[1]**	**C. Conversion to 2022-$I[2]**	**D. Conversion to $USD[3]**
Au Yeung et al. [4]	2012	United States	USD-2011	USD	1.00	1.29	1.00	1.00
Bahrainwala et al. [5]	2020	Madagascar	USD-2019	MGA	3618.32^	1.21	1199.19	4096.12
Beeler Asay et al. [6]	2020	United States	USD-2017	USD	1.00	1.18	1.00	1.00
Broomhead & Mars [7]	2012	South Africa	USD-2005	ZAR	6.60	2.68	6.98	16.36
Buchman & Cabello [8]	2017	United States	USD-2017*	USD	1.00	1.18	1.00	1.00
Daftary et al. [9]	2017	Ethiopia	USD-2017*	ETB	23.87^	2.35	17.03	51.76
Fekadu et al. [10]	2021	United States	USD-2021	USD	1.00	1.07	1.00	1.00
Garfein et al. [11]	2018	United States	USD-2017	USD	1.00	1.18	1.00	1.00
Gashu et al. [12] (pre-print)	2021	Ethiopia	USD-2021*	ETB	15.00	1.31	17.03	51.76
Guo et al. (A) [13]	2020	China	CNY-2020*	CNY	1.00	1.08	4.10	6.74
Guo et al. (B) [14]	2020	China	CNY-2020*	CNY	1.00	1.08	4.10	6.74
Holzman et al. [15]	2018	United States	USD-2018*	USD	1.00	1.15	1.00	1.00
Krueger et al. [16]	2010	United States	USD-2010*	USD	1.00	1.32	1.00	1.00
Lam et al. [17]	2019	United States	USD-2016	USD	1.00	1.20	1.00	1.00
Louwagie et al. [18]	2022	South Africa	USD-2019	ZAR	14.45	1.18	6.98	16.36
Manyazewal et al. [19]	2022	Ethiopia	ETB-2021	ETB	1.00	1.31	17.03	51.76
Mukora et al. [20] (abstract)	2022	South Africa	USD-2022*	ZAR	16.45	1.00	6.98	16.36
Nsengiyumva et al. [21]	2018	Brazil	USD-2016	BRL	3.49^	1.43	2.56	5.16
Nsengiyumva et al. [22] (pre-print)	2023	Bangladesh	USD-2022	BDT	91.75^	1.00	32.11	91.75
		Haiti	USD-2022	HTG	115.63^	1.00	55.74	115.63
		Moldova	USD-2022	MDL	18.90^	1.00	6.64	18.90
		Philippines	USD-2022	PHP	54.48^	1.00	18.78	54.48
		Tanzania	USD-2022	TZS	2300.58^	1.00	853.89	2300.58[&]
Peng et al. [23] (abstract)	2014	China	USD-2014*	CNY	6.14^	1.21	4.10	6.74
Ravenscroft et al. [24]	2020	Moldova	MDL-2020*	MDL	1.00	1.22	6.64	18.90
Saha et al. [25]	2022	India	USD-2020	INR	79.58	1.13	20.14	78.60
Salcedo et al. [26]	2021	United States	USD-2017	USD	1.00	1.18	1.00	1.00
Siddiqui et al. [27]	2019	United States	USD-2014[&]	USD	1.00	1.22	1.00	1.00
Story et al. [28]	2019	UK	GBP-2016	GBP	1.00	1.18	0.67	0.81
Thompson et al. [3]	2022	Uganda	USD-2019	UGX	3704.00	1.12	1298.23	3689.82
Wade et al. [2]	2012	Australia	AUD-2012*	AUD	1.00	1.28	1.51	1.44
Waswa et al. [29] (abstract)	2022	Uganda	USD-2021	UGX	3587.05^	1.07	1298.23	3689.82
Yang et al. [30]	2022	Morocco	USD-2018	MAD	9.12	1.11	3.82	10.16

Notes: Total adjustment factor (PPP - $I) = A * B * C. Total adjustment factor ($USD) = A * B * D

1. Inflation in local currency using GDP implicit deflator from IMF [31] from the currency year to 2022

2. Conversion using PPP from IMF dataset [31]

3. Using average exchange rates for 2022 as reported by the IMF [32]. [&]Average of first 8 months of 2022 since entire period was unavailable for Tanzania

*Currency year assumed to be publication year, ^Exchange rate to local currency not explicitly reported. Average market exchange rates for the currency year used (obtained from IMF dataset [32]) [&]Not explicitly reported but referenced cost inputs from 2014.

S4 Supplemental results

Table A3: Summary of included costs in each full text study

		Provider costs						Patient Costs	
**Study**	**DATs assessed**	**Staff**	**TB drugs & treatment**	**Travel & supplies**	**Fixed assets & technology**	**Implement-ation**	**Overhead**	**Out-of-pocket**	**Indirect**
Bahrainwala et al. (2020)	Digital Pillboxes	☑	☑	✓	☑	✓	☑		
Broomhead & Mars (2012)	Digital Pillboxes	☑	☑		☑	✓			
Manyazewal et al. (2022)	Digital Pillboxes							☑	☑
Yang et al (2022)	Digital Pillboxes	☑	☑	☑	☑	✓	☑		
Saha et al. (2022)	Digital Pillboxes	☑		☑	☑	☑			
Au Yeung et al. (2012)	Ingestible Sensors	☑	☑	✓	☑			✓	☑
Nsengiyumva et al. (2018)	Multiple	☑	☑	☑	✓	✓	✓	☑	☑
Nsengiyumva et al. (2023)	Multiple	☑		☑	☑	☑			
Daftary et al. (2017)	Phone-based			☑					
Thompson et al. (2022)	Phone (Pill sleeve)	☑		☑	☑	☑	☑		
Gashu et al. (2021)	SMS-Based Only			✓					
Louwagie et al. (2022)	SMS-Based Only	☑	☑		✓	☑	✓		
Beeler Asay et al. (2020)	VOT	☑		☑	✓	✓		☑	☑
Buchman & Cabello (2017)	VOT	☑		☑	✓				
Fekadu et al. (2021)	VOT	☑	☑	✓	✓				
Garfein et al. (2018)	VOT	☑		☑	✓				
Guo et al. (A) (2020)	VOT	Unclear		✓	✓				
Guo et al. (B) (2020)	VOT			✓					
Holzman et al (2018)	VOT	☑		☑	☑				
Krueger et al. (2010)	VOT	☑		✓					
Lam et al. (2019)	VOT	☑		☑	✓				
Ravenscroft et al. (2020)	VOT							✓	
Salcedo et al. (2021)	VOT	☑	☑	☑	☑			☑	
Siddiqui et al. (2019)	VOT	☑		☑	✓				
Story et al. (2019)	VOT	☑		☑	☑				
Wade et al. (2012)	VOT	☑		☑	☑		☑		
**Total studies considering category:**		**20/26**	**8/26**	**22/26**	**20/26**	**9/26**	**6/26**	**6/26**	**4/26**

☑Category included in costs. ✓Only one item from category included in costs.

Table A4: Provider costs for VOT and standard of care (SoC) for studies with multiple sites (see ***Error! Reference source not found.****2 in main text)*

Study	Country	Site	DAT pts.	VOT Type	Duration	SOC	Provider costs per patient VOT	SoC	Incremental	Costs included
Beeler Asay et al. (2020)	USA	NYC	186/year	Async.	6mo – Calc.	DOT (Field)	$1,394	$3,375	-$1,980	S, T, F, I
		NYC	186/year	Async.	6mo – Calc.	DOT (Clinic)	$1,394	$1,054	$340	
		NYC	399/year	Sync.	6mo – Calc.	DOT (Field)	$1,208	$3,375	-$2,167	
		NYC	399/year	Sync.	6mo – Calc.	DOT (Clinic)	$1,208	$1,054	$154	
		RI	9/year	Async.	6mo – Calc.	DOT (Field)	$4,624	$3,139	$1,485	
		SF	62/year	Async.	6mo – Calc.	DOT (Field)	$1,491	$2,449	-$959	
		SF	62/year	Async.	6mo – Calc.	DOT (Clinic)	$1,491	$3,942	-$2,452	
Krueger et al. (2010)	USA	Pierce	41	Sync.*	5mo	DOT (Field)	Incremental only		-$3,171	S, T
		Squamish	16	Sync.*	5mo	DOT (Field)	Incremental only		-$3,373	

Note: Beeler Asay et al. did not provide a specific site breakdown on the number of patients that made up the combined sample of 63 patients for asynchronous and 57 patients for synchronous VOT. They did provide however annual patient volumes for each site.
*Study considers savings in patients that started on DOT and were switched to VOT for variable portions of their treatment (average of 20 weeks on VOT for all patients)

Beeler Asay et al. had 3 study sites in the United States described as New York City, Rhode Island and San Francisco. The Rhode Island site had a much smaller case load than the other sites and so fixed software costs for asynchronous VOT were proportionally much higher and represented over 80% of the per patient cost. Societal costs were not broken down by site for this study. While costs were presented in the study on a per session basis, the costs were converted to per patient using the process described in *Methods*.

Table A5: Provider costs for VOT and DOT on a per observation basis

Study	Country	DAT pts.	VOT Type	SOC	Provider costs per observation			Costs included
					**VOT**	**SoC**	**Incremental**	
Beeler Asay et al. (2020)	USA	63	Async.	DOT (Field)	$13.58	$22.22	-$8.64	S, T, F, I
				DOT (Clinic)	$13.58	$14.27	-$0.69	
		50	Sync.	DOT (Field)	$6.64	$22.22	-$15.58	
				DOT (Clinic)	$6.64	$14.27	-$7.64	
Fekadu et al. (2021)	USA	NR++	Sync.	DOT (Clinic)	$79.93	$125.40	-$45.47	S, D, T, F
Garfein et al. (2018)	USA	225	Async.	DOT (Field)	$22.57	$42.68	-$20.11	S, T, F
Guo et al. (B) (2020)	China	90	Async.	DOT (Clinic)	$0.14	$1.05	-$0.91	T (Transport only^)
Holzman et al (2018)	USA	15/vehicle±	Async.	DOT (Field)	$5.96	$18.26	-$12.30	S, T, F
Krueger et al. (2010)	USA	57	Sync.	DOT (Field)	Incremental		-$41.62	S, T
Lam et al. (2019)	USA	81	Sync.	DOT (Clinic)	$7.84	$10.14	-$2.30	S, T, F
				DOT (Field)	$7.84	$23.78	-$15.94	
		41	Async.	DOT (Clinic)	$6.41	$10.14	-$3.73	
				DOT (Field)	$6.41	$23.78	-$17.36	
Nsengiyumva et al. (2018)	Brazil	NR++ – DS	Sync.	DOT (Clinic)	$14.19	$23.27	-$9.08	S, D, T, F, I, O
		NR++ – DR	Sync.	DOT (Clinic)	$93.24	$104.98	-$11.75	
Nsengiyumva et al. (2023)	Moldova	173 – DS	Async.	DOT (Clinic)	$4.81 ($2.72)	$7.97	-$3.16 (-$5.25)	S, T, F, I
		135 – DR	Async.	DOT (Clinic)	$4.81 ($2.72)	$7.97	-$3.16 (-$5.25)	
	Haiti	87	Async.	DOT (Clinic)	$13.30 ($12.51)	$7.16	$6.14 ($5.35)	
	Philippines	119	Async.	DOT (Clinic)	$7.10 ($5.32)	$0.27	$6.83 ($5.05)	
Siddiqui et al. (2019)	USA	47	Async.	DOT (Field)	$24.32	$42.85	-$18.53	S, T, F
Story et al. (2019)	UK	50§	Sync.	DOT (Clinic)	$15.87	$77.00	-$61.13	S, T, F
Wade et al. (2012)	Australia	47§	Sync**	DOT (Field)	$15.94	$23.84	-$7.89	S, T, F, O

Costs included: S = Staff, D = Drugs and Treatment, T = Travel and supplies, F = Fixed assets and technology, I = Implementation, O = Overhead, P = Patient expenses
DS = Drug susceptible, DR = Drug resistant. Cost in parentheses are calculated with fixed costs annuitized over a 5-year useful life.
§These studies observed costs from 112 patients (Story et al.) and 58 patients (Wade et al.) but modelled clinics with 50 and 47 patients respectively as base cases. These studies also provided multiple scenarios with varying patients per clinic not shown here. 5X/week DOT is shown for Story et al.
^Patients given funds to cover cost of roundtrip to clinic on public transportation
±This study observed costs from 28 patients but modelled a clinic assuming 15 patients per vehicle used in field DOT. ++These patients were a modelled cohort.
**Study considers 5% of patients remaining on DOT even in the DAT case

Table A6: Provider costs for synchronous and asynchronous VOT on a per observation basis

Study	Country	Async. Pts.	Sync. Pts.	Setting	Provider costs per observation			Costs Included
					**Asynchronous**	**Synchronous**	**Incremental**	
Lam et al. (2019)	USA	81	41	**New York City**	**$6.41**	**$7.84**	**-$1.43**	S, T, F
Beeler Asay et al. (2020)	USA	257/year	399/year	All sites	$13.58	$6.64	$6.94	S, T, F, I
		186/year	399/year	**New York City**	**$7.66**	**$6.64**	**$1.02**	
		62/year	-	San Francisco	$8.19	-		
		9/year	-	Rhode Island	$25.40	-		

Costs included: S = Staff, D = Drugs and Treatment, T = Travel and supplies, F = Fixed assets and technology, I = Implementation, O = Overhead, P = Patient expenses
Note: Beeler Asay et al. did not report a site breakdown on the number of patients that made up the combined sample of 63 patients for asynchronous and 57 patients for synchronous VOT. They provided annual patient volumes for each site.

Table A7: Incremental cost per DALY averted from provider and societal perspectives

						ICER - $/DALY averted [#]		
**Study**	**Country**	**DAT pts.**	**DAT used**	**Intervention Duration**	**SoC**	**Health system**	**Societal**	**Costs included**
Bahrainwala et al. (2020)	Madagascar	276[++]	Digital pillbox	6mo	DOT	$124[§]		S, D, T, F, I, O
		445[++]		6mo	SAT	$76[§]		
Fekadu et al. (2021)	United States	NR[++]	VOT	6mo	DOT	DAT Dominant[§]		S, D, T, F
		NR[++]		6mo	SAT	DAT Dominant[&]		
Nsengiyumva et al. (2018)	Brazil	NR[++]	99DOTS	9mo – TBI[C]	SAT	$410<br>($20 to $869)	$1,029<br>(-$195 to $2647)	S, D, T, F, I, O, (P)
		NR[++]		9mo – TBI[G]	SAT	$2,674<br>($1247 to $3609)	$5,721<br>($1569 to $10752)	
		NR[++]	SMS + reply	9mo – TBI[C]	SAT	$240<br>(-$111 to $607)	$859<br>(-$328 to $2,387)	
		NR[++]		9mo – TBI[G]	SAT	$1,952<br>($748 to $2,887)	$4,999<br>($1,044 to $10,084)	
		NR[++]	VOT (sync.)	9mo – TBI[C]	SAT	$19,139<br>($7,140 to $34,347)	$19,758<br>($7,281 to $34,252)	
		NR[++]		9mo – TBI[G]	SAT	$82,923<br>($40,082 to $118,490)	$85,970<br>($40,965 to $121,906)	
		NR[++]	Digital pillbox	9mo – TBI[C]	SAT	$2,299<br>($732 to $4,238)	$2,918<br>($759 to $5,468)	
		NR[++]		9mo – TBI[G]	SAT	$10,775<br>($5,313 to $15,261)	$13,822<br>($6,012 to $20,418)	
Yang et al. (2022)	Morocco	NR[++]	Digital pillbox	6mo*	DOT+SAT	$1,146[&&]		S, D, T, F, I, O

Costs included: S = Staff, D = Drugs and Treatment, T = Travel and supplies, F = Fixed assets and technology, I = Implementation, O = Overhead, P = Patient expenses
TBI = TB infection ([C] Cohort modelled based on persons who were close contacts of persons with contagious TB disease. [G] Cohort modelled as unselected persons with TBI).
[++]These patients were a modelled cohort. [#]Negative values indicate cost savings. Values in parentheses are 95% uncertainty intervals
*The model considers most patients as drug-sensitive (6 months) however patients on retreatment are modelled to be treated for 8 months, and MDR patients for 24 months
§Calculated from available data in sensitivity analyses and therefore no uncertainty range available
[&]95% uncertainty range via probabilistic sensitivity analysis: Reduction in DALYs (0.4385-0.0.4440) and reduction in cost ($1,924-$2,082)
[&&]95% uncertainty range via probabilistic sensitivity analysis: Reduction in DALYs (0.08-0.32), increased cost ($246-417)

Table A8: Incremental cost per TB case averted with provider and societal perspectives.

Study	Country	DAT pts.	DAT used	Intervention Duration	SoC	ICER - $/case averted[#] Provider	ICER - $/case averted[#] Societal	Costs included
Nsengiyumva et al. (2018)	Brazil	NR[++]	99DOTS	9mo – TBI[C]	SAT	$2,026 (-$78 to $4,285)	$5,091 (-$1,101 to $12,520)	S, D, T, F, I, O, (P)
		NR[++]		9mo – TBI[G]	SAT	$12,063 ($5,647 to $16,149)	$25,641 ($7,136 to $48,074)	
		NR[++]	SMS + reply	9mo – TBI[C]	SAT	$1,193 (-$533 to $3,037)	$4,255 (-$1,599 to $11,394)	
		NR[++]		9mo – TBI[G]	SAT	$8,751 ($3,426 to $12,946)	$22,407 ($4,698 to $44,640)	
		NR[++]	VOT (sync.)	9mo – TBI[C]	SAT	$94,771 ($36,588 to $163,522)	$97,832 ($36,612 to $168,051)	
		NR[++]		9mo – TBI[G]	SAT	$371,689 ($180,731 to $529,220)	$385,345 ($188,498 to $544,358)	
		NR[++]	Digital pillbox	9mo – TBI[C]	SAT	$11,392 ($3,660 to $20,894)	$14,453 ($3,545 to $26,307)	
		NR[++]		9mo – TBI[G]	SAT	$48,302 ($24,062 to $68,704)	$61,956 ($27,552 to $88,431)	
Yang et al. (2022)	Morocco	NR[++]	Digital pillbox	6mo*	DOT+SAT	$1,834[§]		S, D, T, F, I, O

Costs included: S = Staff, D = Drugs and Treatment, T = Travel and supplies, F = Fixed assets and technology, I = Implementation, O = Overhead, P = Patient expenses
TBI = TB infection ([C] Cohort modelled based on persons who were close contacts of persons with contagious TB disease. [G] Cohort modelled as unselected persons with TBI).
[++]These patients were a modelled cohort. [#]Negative values indicate cost savings. Values in parentheses are 95% uncertainty intervals
*The model considers most patients as drug-sensitive (6 months) however patients on retreatment are modelled to be treated for 8 months, and MDR patients for 24 months
[#]Negative values indicate cost savings. Values in parentheses are 95% uncertainty ranges. [§]Calculated from available data and therefore an uncertainty range is not available.

Table A9: Incremental cost per QALY gained with provider and societal perspectives

Study	Country	DAT pts.	DAT used	Intervention Duration	SoC	ICER - $/QALY gained Provider	ICER - $/QALY gained Societal	Costs included
Louwagie et al. (2022)	South Africa	122	SMS + motivational interviewing	3mo	NR	$36,248 - QALYs gained not significant		S, D, F, I, O
Saha et al. (2022)	India	200[++]	Digital pillbox	6mo	DOT (NR)	$655[&]		S, T, F, I
Salcedo et al. (2021)	USA	100[§]	VOT – Async. (AI)	8mo*	DOT (Clinic & Field)		DAT Dominant	S, D, T, F, P

Costs included: S = Staff, D = Drugs and Treatment, T = Travel and supplies, F = Fixed assets and technology, I = Implementation, O = Overhead, P = Patient expenses
§Data inputs to model were obtained from 43 patients receiving the DAT and 73 receiving the SoC. The model then compared a cohort of 100 patients in each arm.
*The model used observed treatment completion probabilities to model successful completion or required prolongation of treatment in each arm at the end of each month (minimum 5 months, maximum 16 months). Mean treatment durations in both groups were 8 months.
[&]Perspective was labelled as societal in study, but all costs reported were for provider
[++]Saha et al. 200 patients received the intervention in a quasi-experimental study, but modeling was used for cost effectiveness based on completion probabilities found in study.

Figure A1: Summary of incremental costs to providers. Each point represents one comparison; some studies evaluated multiple scenarios (e.g. different comparators, TB disease versus TB infection, etc.)

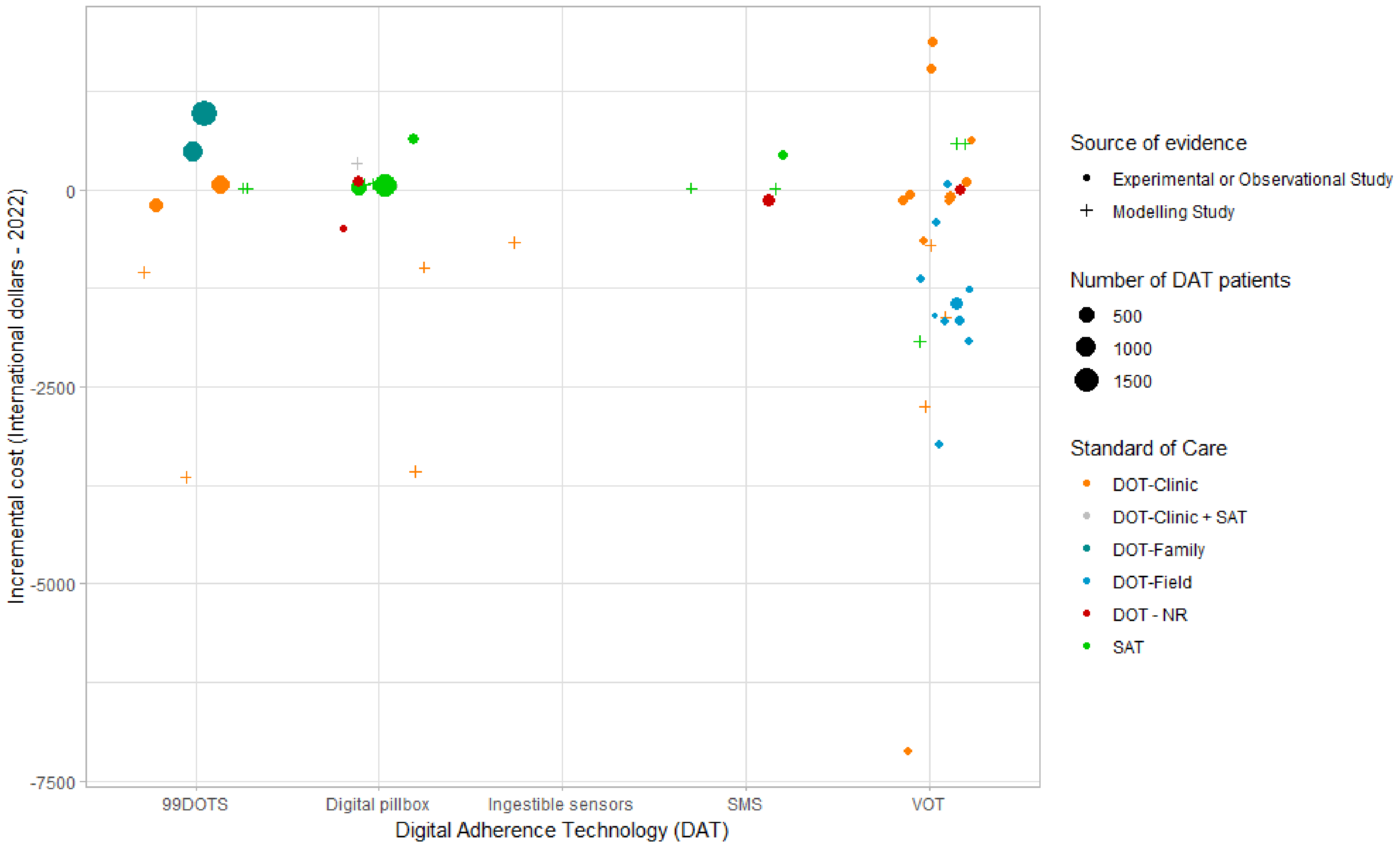

S5 USD results – Sensitivity analysis

Table A3: Provider costs for VOT and standard of care (SoC) – USD based on market exchange rates.

Study	Country	DAT pts.	VOT Type	Duration	SOC	Provider costs per person treated			Costs included
						VOT	SoC	Incremental	
Beeler Asay et al. (2020)	USA	63	Async.	6mo – Calc.	DOT (Field)	$2,471	$2,888	-$417	S, T, F, I
				6mo – Calc.	DOT (Clinic)	$2,471	$1,856	$616	
		50	Sync.	6mo – Calc.	DOT (Field)	$1,208	$2,888	-$1,680	
				6mo – Calc.	DOT (Clinic)	$1,208	$1,856	-$648	
Buchman and Cabello (2017)	USA	24	Sync.	NR	DOT (Field)	Incremental		-$1,256	S, T, F
Fekadu et al. (2021)	USA	NR[++]	Sync.	6mo	SAT	$13,428	$15,353	-$1,925	S, D, T, F
				6mo	DOT (Clinic)	$13,428	$15,048	-$1,620	
Garfein et al. (2018)	USA	225	Async.	6mo	DOT (Field)	$4,108	$5,549	-$1,441	S, T, F
Guo et al. (A) (2020)	China	199	Sync.	NR	DOT (NR)	$5	$11	-$6	S, T, F
Guo et al. (B) (2020)	China	90	Async.	6mo	DOT (Clinic)	$8	$44	-$36	T (Transport only^)
Holzman et al (2018)	USA	15/vehicle±	Async.	6mo	DOT (Field)	$775	$2,373	-$1,599	S, T, F
Krueger et al. (2010)	USA	57	Sync.	5mo	DOT (Field)	Incremental		-$3,228	S, T
Lam et al. (2019)	USA	81	Sync.	6mo – Calc.	DOT (Clinic)	$1,427	$1,319	$108	S, T, F
				6mo – Calc.	DOT (Field)	$1,427	$3,091	-$1,664	
		41	Async.	6mo – Calc.	DOT (Clinic)	$1,168	$1,319	-$151	
				6mo – Calc.	DOT (Field)	$1,168	$3,091	-$1,923	
Nsengiyumva et al. (2018)	Brazil	NR[++]	Sync.	6mo – DS	DOT (Clinic)	$548	$898	-$351	S, D, T, F, I, O
				18mo – DR	DOT (Clinic)	$10,795	$12,155	-$1,360	
				9mo – TBI[G]	SAT	$344	$58	$286	
				9mo – TBI[C]	SAT	$337	$48	$289	
Nsengiyumva et al. (2023)	Moldova	173	Async.	6mo – DS	DOT (Clinic)	$172 ($304)	$336	-$164 (-$32)	S, T, F, I
		135	Async.	9mo – DR	DOT (Clinic)	$259 ($457)	$504	-$245 (-$47)	
	Haiti	87	Async.	6mo – DS	DOT (Clinic)	$1,085 ($1,154)	$414	$671 ($740)	
	Philippines	119	Async.	9mo - DR	DOT (Clinic)	$495 ($661)	$17	$478 ($644)	
Siddiqui et al. (2019)	USA	47	Async.	6mo – Calc.	DOT (Field)	$4,426	$5,570	-$1,145	S, T, F
Story et al. (2019)	UK	50[§]	Sync.	6mo	DOT (Clinic)	$2,400	$8,316	-$5,916	S, T, F
Wade et al. (2012)	Australia	47[§]	Sync*	5mo	DOT (Field)	$2,350	$2,293	$58	S, T, F, O

Costs included: S = Staff, D = Drugs and Treatment, T = Travel and supplies, F = Fixed assets and technology, I = Implementation, O = Overhead, P = Patient expenses
Calc. = Converted from per observation cost (see Methods). DS = Drug susceptible, DR = Drug resistant., Async. asynchronous, Sync. Synchronous, NR not reported. TBI = TB infection (C Cohort modelled based on persons who were close contacts of persons with contagious TB disease. G Cohort modelled as unselected persons with TBI).
Cost in parentheses are calculated with fixed costs annuitized over a 5-year useful life.
^Patients given funds to cover cost of roundtrip to clinic on public transportation §These studies observed costs from 112 patients (Story et al.) and 58 patients (Wade et al.) but modelled clinics with 50 and 47 patients respectively as base cases. These studies also provided multiple scenarios with varying patients per clinic not shown here. 5X/week DOT is shown for Story et al.
±This study observed costs from 28 patients but modelled a clinic assuming 15 patients per vehicle used in field DOT
[++]These patients were a modelled cohort. *Study considers 5% of patients remaining on DOT even in the DAT case.

Table A4: Societal (or patient-only) costs for video-observed therapy (VOT) and standard of care (SoC) – USD based on market exchange rates.

						Costs per person treated			
**Study**	**Country**	**DAT pts.**	**VOT Type**	**Duration**	**SOC**	**VOT**	**SoC**	**Incremental**	**Costs included**
Beeler Asay et al. (2020)	USA	63	Async.	6mo	DOT (Field)	$2,707	$7,053	-$4,347	S, T, F, I, P
				6mo	DOT (Clinic)	$2,707	$3,274	-$567	
		50	Sync.	6mo	DOT (Field)	$1,424	$7,053	-$5,629	
				6mo	DOT (Clinic)	$1,424	$3,274	-$1,849	
Nsengiyumva et al. (2018)	Brazil	NR[++]	Sync.	6mo – DS	DOT (Clinic)	$693	$1,206	-$513	S, D, T, F, I, O, P
				18mo – DR	DOT (Clinic)	$11,250	$13,183	-$1,934	
				9mo – TBI[G]	SAT	$461	$161	$299	
				9mo – TBI[C]	SAT	$469	$175	$295	
Ravenscroft et al. (2020)	Moldova	155	Async.	4mo	DOT (Clinic)	$12	$45	-$33	P (Transport only)
Salcedo et al. (2021)	USA	100[§]	Async. (AI)	8mo[#]	DOT (Clinic & Field)	$3,139	$5,759	-$2,619	S, D, T, F, P

Costs included: S = Staff, D = Drugs and Treatment, T = Travel and supplies, F = Fixed assets and technology, I = Implementation, O = Overhead, P = Patient expenses
AI artificial intelligence, Async. asynchronous, Sync. Synchronous, NR not reported.
TBI = TB infection (C Cohort modelled based on persons who were close contacts of persons with contagious TB disease. G Cohort modelled as unselected persons with TBI).
[++]These patients were a modelled cohort.
§Data inputs to model were obtained from 43 patients receiving the DAT and 73 receiving the SoC. The model then compared a cohort of 100 patients in each arm.
[#]The model used observed treatment completion probabilities to model successful completion or required prolongation of treatment in each arm at the end of each month (minimum 5 months, maximum 16 months). Mean treatment durations in both groups were 8 months.

Table A5: Provider and societal costs for digital pillbox intervention and standard of care (SoC) – USD based on market exchange rates

Study	Country	DAT pts.	Co-intervention	Duration	SOC	Provider costs per person treated: DAT	SoC	Incremental	Costs included
Bahrainwala et al. (2020)	Madagascar	475++	-	6 mo	SAT	$1,404	$1,395	$9	S, D, T, F, I, O
		276++	DOT (Clinic) + SAT	6 mo	DOT (Clinic) + SAT	$560	$545	$15	
Broomhead & Mars (2012)	S. Africa	24++	DOT (NR)	6 mo	DOT alone (NR)	$762	$969	-$207	S, D, F, I
Mukora et al. (2022)	S. Africa	1305^	-	6 mo	SAT	$50	$29	$22	Abstract only
Nsengiyumva et al. (2018)	Brazil	NR++	-	6 mo – DS	DOT (Clinic)	$409	$898	-$490	S, D, T, F, I, O
				18 mo – DR	DOT (Clinic)	$10,386	$12,155	-$1,768	
				9 mo – TBI[G]	SAT	$86	$48	$38	
				9 mo – TBI[C]	SAT	$93	$58	$35	
Saha et al. (2022)	India	200	-	6 mo	DOT (NR)	$95	$69	$26	S, T, F, I
Yang et al. (2022)	Morocco	206	-	6 mo	SAT	$396	$155	$241	S, D, T, F, I, O
		NR±	-		DOT + SAT*	$740	$615	$125	
						**Societal costs per person treated**			
Manyazewal et al. (2022)	Ethiopia	52	-	2 mo	DOT (Clinic)	$0.61	$11	-$10	P
Nsengiyumva et al. (2018)	Brazil	NR++	-	6 mo – DS	DOT (Clinic)	$554	$1,206	-$652	S, D, T, F, I, O, P
				18 mo – DR	DOT (Clinic)	$10,841	$13,183	-$2,342	
				9 mo – TBI[G]	SAT	$209	$161	$47	
				9 mo – TBI[C]	SAT	$218	$175	$43	

Costs included: S = Staff, D = Drugs and Treatment, T = Travel and supplies, F = Fixed assets and technology, I = Implementation, O = Overhead, P = Patient expenses).

DS = Drug susceptible, DR = Drug resistant, TBI = TB infection ([C] Cohort modelled based on persons who were close contacts of persons with contagious TB disease. [G] Cohort modelled as unselected persons with TBI).

DOT directly observed therapy, SAT self-administered therapy, NR not reported.

++These patients were a modelled cohort. For Broomhead & Mars, while the 24 patients did receive the intervention, the costs were entirely modelled using literature values. For Bahrainwala et al. the costs are presented per diagnosed patient only (to ensure comparability between other studies)

±This study observed costs from an implementation of 206 patients but then modelled costs in a separate scenario considering costs of retreatment, MDR development & treatment, etc.

^ Target number of patients from protocol of parent RCT [1]. Actual patient numbers not reported in this abstract

*2 months of DOT (facility details not reported) followed by 4 months of SAT. The model considers most patients as drug-sensitive (6-month treatment course) however patients on retreatment are modelled to be treated for 8 months, and MDR patients for 24 months.

Table A6: Provider and societal costs for SMS-based interventions and standard of care (SoC) – USD based on market exchange rates

Study	Country	DAT pts.	Co-intervention	Duration	SOC	**Provider costs per person treated** DAT	SoC	Incremental	Costs included
Gashu et al. (2021)	Ethiopia	131*	DOT (Family)	4mo	N/A	$0.16			T (Airtime only)
Louwagie et al. (2022)	S. Africa	122	Motivational interviewing	3mo	SAT	$236	$51	$185	S, D, F, I, O
Nsengiyumva et al. (2018)	Brazil	NR[++]	Patient reply	9mo – TBI[G]	SAT	$57	$48	$9	S, D, T, F, I, O
				9mo – TBI[C]	SAT	$63	$58	$5	
Peng et al. (2014)	China	234	-	6mo	DOT (Type not specified)	$43	$129	-$86	Abstract only
						**Societal costs per person treated**			
Nsengiyumva et al. (2018)	Brazil	NR[++]	Patient Reply	9mo – TBI[G]	SAT	$184	$161	$22	S, D, T, F, I, O, P
				9mo – TBI[C]	SAT	$191	$175	$16	

Costs included: S = Staff, D = Drugs and Treatment, T = Travel and supplies, F = Fixed assets and technology, I = Implementation, O = Overhead, P = Patient expenses
Calc. = Converted from per day cost (see Methods). TBI = TB infection ([C] Cohort modelled based on persons who were close contacts of persons with contagious TB disease. [G] Cohort modelled as unselected persons with TBI).
*Target number of patients from protocol of parent RCT. Actual patient numbers not reported in this pre-print
[++]These patients were a modelled cohort.

Table A7: Provider and societal costs per person for phone-based with medication sleeves intervention ("99DOTS") – USD based on market exchange rates

Study	Country	DAT pts.	Duration	SoC	Provider costs per person treated: DAT	SoC	Incremental	Costs included
Nsengiyumva et al. (2018)	Brazil	NR++	6mo – DS	DOT (Clinic)	$381	$898	-$518	S, D, T, F, I, O
			18mo – DR	DOT (Clinic)	$10,348	$12,155	-$1,806	
			9mo – TBI[G]	SAT	$58	$48	$10	
			9mo – TBI[C]	SAT	$65	$58	$7	
Nsengiyumva et al. (2023)	Tanzania	976	6mo	DOT (Family)	$163 ($174)	$0.00	$163 ($174)	S, T, F, I
	Bangladesh	719	6mo	DOT (Clinic)	$81 ($98)	$74	$7 ($24)	
	Philippines	396	6mo	DOT (Clinic)	$79 ($106)	$176	-$97 (-$70)	
Thompson et al. (2022)	Uganda	1800/year§	6mo#	DOT (Family)	$339 ($58)	$0	$339 ($58)	S, T, F, I, O
Waswa et al (2022)	Uganda	1086	6mo – Calc.	None	$27			Abstract only*
					**Societal costs per person treated**			
Nsengiyumva et al. (2018)	Brazil	NR++	6mo – DS	DOT (Clinic)	$526	$1,206	-$680	S, D, T, F, I, O, P
			18mo – DR	DOT (Clinic)	$10,803	$13,183	-$2,380	
			9mo – TBI[G]	SAT	$181	$161	$19	
			9mo – TBI[C]	SAT	$190	$175	$15	

Costs included: S = Staff, D = Drugs and Treatment, T = Travel and supplies, F = Fixed assets and technology, I = Implementation, O = Overhead, P = Patient expenses
DS = Drug susceptible, DR = Drug resistant, TBI = TB infection ([C] Cohort modelled based on persons who were close contacts of persons with contagious TB disease. [G] Cohort modelled as unselected persons with TBI). Cost in parentheses are calculated with fixed costs annuitized over a 5-year useful life.
§This study observed detailed resource unit costs from five clinics (with 81 patients) in parent RCT and then applied these costs to resource estimates from all 18 clinics in the RCT. They used these observed costs (from 891 intervention patients) and applied them to a modelled clinic with a service volume of 1800 patients per year.
#Intervention was 6 months but costs account for patients being lost to follow up. ++These patients were a modelled cohort.
*Costs described as "running costs only" (i.e. excluding start-up costs)

Table A8: Provider and societal costs per treatment course for other DAT interventions – USD based on market exchange rates

Study	Country	DAT pts.	DAT used	Duration	SOC	Provider costs per person treated			Costs included
						DAT	SoC	Incremental	
Au Yeung et al. (2012)	USA	NR++	Ingestible sensors	4 mo	DOT (Clinic)*	$1,618	$2,289	-$671	S, D, T, F
Daftary et al. (2017)	Ethiopia	2300 (approx.)	Interactive Voice Calls	6 mo - IPT	N/A	$49	-	-	T
						**Societal costs per person treated**			
Au Yeung et al. (2012)	USA	NR++	Ingestible sensors	4 mo	DOT (Clinic)*	$1,680	$3,030	-$1,350	S, D, T, F, P

Costs included: S = Staff, D = Drugs and Treatment, T = Travel and supplies, F = Fixed assets and technology, I = Implementation, O = Overhead, P = Patient expenses
IPT = Isoniazid preventative therapy. *3X/week DOT is shown for Au Yeung et al. ++These patients were a modelled cohort.

Table A9: Incremental cost per DALY averted from provider and societal perspectives -USD based on market exchange rates

Study	Country	DAT pts.	DAT used	Intervention Duration	SoC	ICER - $/DALY averted # Health system	Societal	Costs included
Bahrainwala et al. (2020)	Madagascar	276++	Digital pillbox	6mo	DOT	$36§		S, D, T, F, I, O
		445++		6mo	SAT	$22§		
Fekadu et al. (2021)	United States	NR++	VOT	6mo	DOT	DAT Dominant§		S, D, T, F
		NR++		6mo	SAT	DAT Dominant&		
Nsengiyumva et al. (2018)	Brazil	NR++	99DOTS	9mo – TBI[C]	SAT	$203 ($10 to $430)	$509 (-$97 to $1,310)	S, D, T, F, I, O, (P)
		NR++		9mo – TBI[G]	SAT	$1,323 ($617 to $1786)	$2,831 ($776 to $5,320)	
		NR++	SMS + reply	9mo – TBI[C]	SAT	$119 (-$55 to $300)	$425 (-$162 to $1,181)	
		NR++		9mo – TBI[G]	SAT	$966 ($370 to $1,428)	$2,473 ($517 to $4,989)	
		NR++	VOT (sync.)	9mo – TBI[C]	SAT	$9,470 ($3,533 to $16,994)	$9,776 ($3,602 to $16,947)	
		NR++		9mo – TBI[G]	SAT	$41,028 ($19,832 to $58,626)	$42,536 ($20,268 to $60,316)	
		NR++	Digital pillbox	9mo – TBI[C]	SAT	$1,138 ($362 to $2,097)	$1,444 ($376 to $2,705)	
		NR++		9mo – TBI[G]	SAT	$5,331 ($2,629 to $7,551)	$6,839 ($2,975 to $10,102)	
Yang et al. (2022)	Morocco	NR++	Digital pillbox	6mo*	DOT+SAT	$431&&		S, D, T, F, I, O

Costs included: S = Staff, D = Drugs and Treatment, T = Travel and supplies, F = Fixed assets and technology, I = Implementation, O = Overhead, P = Patient expenses
TBI = TB infection ([C] Cohort modelled based on persons who were close contacts of persons with contagious TB disease. [G] Cohort modelled as unselected persons with TBI).
++These patients were a modelled cohort. #Negative values indicate cost savings. Values in parentheses are 95% uncertainty intervals
*The model considers most patients as drug-sensitive (6 months) however patients on retreatment are modelled to be treated for 8 months, and MDR patients for 24 months
§Calculated from available data in sensitivity analyses and therefore no uncertainty range available
&95% uncertainty range via probabilistic sensitivity analysis: Reduction in DALYs (0.4385-0.0.4440) and reduction in cost ($1,924-$2,082)
&&95% uncertainty range via probabilistic sensitivity analysis: Reduction in DALYs (0.08-0.32), increased cost ($92-157)

Table A10: Incremental cost per TB case averted with provider and societal perspectives. – USD based on market exchange rates

Study	Country	DAT pts.	DAT used	Intervention Duration	SoC	ICER - $/case averted [#] Provider	ICER - $/case averted [#] Societal	Costs included
Nsengiyumva et al. (2018)	Brazil	NR[++]	99DOTS	9mo – TBI[C]	SAT	$1,002 (-$39 to $2,120)	$2,519 (-$545 to $6,195)	S, D, T, F, I, O, (P)
		NR[++]		9mo – TBI[G]	SAT	$5,969 ($2,794 to $7,990)	$12,687 ($3,531 to $23,786)	
		NR[++]	SMS + reply	9mo – TBI[C]	SAT	$590 (-$264 to $1,503)	$2,105 (-$791 to $5,637)	
		NR[++]		9mo – TBI[G]	SAT	$4,330 ($1,695 to $6,405)	$11,086 ($2,325 to $22,087)	
		NR[++]	VOT (sync.)	9mo – TBI[C]	SAT	$46,890 ($18,103 to $80,907)	$48,405 ($18,114 to $83,147)	
		NR[++]		9mo – TBI[G]	SAT	$183,902 ($89,421 to $261,845)	$190,659 ($93,264 to $269,334)	
		NR[++]	Digital pillbox	9mo – TBI[C]	SAT	$5,636 ($1,811 to $10,338)	$7,151 ($1,754 to $13,016)	
		NR[++]		9mo – TBI[G]	SAT	$23,899 ($11,905 to $33,993)	$30,654 ($13,632 to $43,753)	
Yang et al. (2022)	Morocco	NR[++]	Digital pillbox	6mo*	DOT+SAT	$690[§]		S, D, T, F, I, O

Costs included: S = Staff, D = Drugs and Treatment, T = Travel and supplies, F = Fixed assets and technology, I = Implementation, O = Overhead, P = Patient expenses
TBI = TB infection ([C] Cohort modelled based on persons who were close contacts of persons with contagious TB disease. [G] Cohort modelled as unselected persons with TBI).
[++]These patients were a modelled cohort. [#]Negative values indicate cost savings. Values in parentheses are 95% uncertainty intervals
*The model considers most patients as drug-sensitive (6 months) however patients on retreatment are modelled to be treated for 8 months, and MDR patients for 24 months
[#]Negative values indicate cost savings. Values in parentheses are 95% uncertainty ranges. [§]Calculated from available data and therefore an uncertainty range is not available.

Table A11: Incremental cost per QALY gained with provider and societal perspectives – USD based on market exchange rates

Study	Country	DAT pts.	DAT used	Intervention Duration	SoC	ICER - $/QALY gained Provider	ICER - $/QALY gained Societal	Costs included
Louwagie et al. (2022)	South Africa	122	SMS + motivational interviewing	3mo	NR	$15,458- QALYs gained not significant		S, D, F, I, O
Saha et al. (2022)	India	200[++]	Digital pillbox	6mo	DOT (NR)	$168[&]		S, T, F, I
Salcedo et al. (2021)	USA	100[§]	VOT – Async. (AI)	8mo*	DOT (Clinic & Field)		DAT Dominant	S, D, T, F, P

Costs included: S = Staff, D = Drugs and Treatment, T = Travel and supplies, F = Fixed assets and technology, I = Implementation, O = Overhead, P = Patient expenses
§Data inputs to model were obtained from 43 patients receiving the DAT and 73 receiving the SoC. The model then compared a cohort of 100 patients in each arm.
*The model used observed treatment completion probabilities to model successful completion or required prolongation of treatment in each arm at the end of each month (minimum 5 months, maximum 16 months). Mean treatment durations in both groups were 8 months.
[&]Perspective was labelled as societal in study, but all costs reported were for provider
[++]Saha et al. 200 patients received the intervention in a quasi-experimental study, but modeling was used for cost effectiveness based on completion probabilities found in study.

One study compared synchronous VOT to field DOT and estimated it would cost $1.17 per additional treatment observation accomplished (95% confidence interval: $0.45-$2.00) to implement VOT in a clinic of 47 patients [2].

Another study compared an intervention involving medication sleeves and phone registration of doses (99DOTS) to family DOT in Uganda, and estimated it would cost $397 per additional treatment success (95% confidence interval: $245-$697) to implement the intervention in a clinic over 5 years [3].

Table A12: Provider costs for VOT and standard of care (SoC) for studies with multiple sites - USD

Study	Country	Site	DAT pts.	VOT Type	Duration	SOC	Provider costs per person treated: VOT	SoC	Incremental	Costs included
Beeler Asay et al. (2020)	USA	NYC	186/year	Async.	6mo – Calc.	DOT (Field)	$1,394	$3,375	-$1,980	S, T, F, I
		NYC	186/year	Async.	6mo – Calc.	DOT (Clinic)	$1,394	$1,054	$340	
		NYC	399/year	Sync.	6mo – Calc.	DOT (Field)	$1,208	$3,375	-$2,167	
		NYC	399/year	Sync.	6mo – Calc.	DOT (Clinic)	$1,208	$1,054	$154	
		RI	9/year	Async.	6mo – Calc.	DOT (Field)	$4,624	$3,139	$1,485	
		SF	62/year	Async.	6mo – Calc.	DOT (Field)	$1,491	$2,449	-$959	
		SF	62/year	Async.	6mo – Calc.	DOT (Clinic)	$1,491	$3,942	-$2,452	
Krueger et al. (2010)	USA	Pierce	41	Sync.*	5mo	DOT (Field)	Incremental		-$3,171	S, T
		Squamish	16	Sync.*	5mo	DOT (Field)	Incremental		-$3,373	

Note: Beeler Asay et al. did not provide a specific site breakdown on the number of patients that made up the combined sample of 63 patients for asynchronous and 57 patients for synchronous VOT. They did provide however annual patient volumes for each site.

*Study considers savings in patients that started on DOT and were switched to VOT for variable portions of their treatment (average of 20 weeks on VOT for all patients)

Beeler Asay et al. had 3 study sites in the United States described as New York City, Rhode Island and San Francisco. The Rhode Island site had a much smaller case load than the other sites and so fixed software costs for asynchronous VOT were proportionally much higher and represented over 80% of the per patient cost. Societal costs were not broken down by site for this study. While costs were presented in the study on a per session basis, the costs were converted to per patient using the process described in *Methods*.

Table A13: Provider costs for VOT and DOT on a per observation basis – USD based on market exchange rates

Study	Country	DAT pts.	VOT Type	SOC	Provider costs per observation			Costs included
					VOT	SoC	Incremental	
Beeler Asay et al. (2020)	USA	63	Async.	DOT (Field)	$13.58	$22.22	-$8.64	S, T, F, I
				DOT (Clinic)	$13.58	$14.27	-$0.69	
		50	Sync.	DOT (Field)	$6.64	$22.22	-$15.58	
				DOT (Clinic)	$6.64	$14.27	-$7.64	
Fekadu et al. (2021)	USA	NR++	Sync.	DOT (Clinic)	$79.93	$125.40	-$45.47	S, D, T, F
Garfein et al. (2018)	USA	225	Async.	DOT (Field)	$22.57	$42.68	-$20.11	S, T, F
Guo et al. (B) (2020)	China	90	Async.	DOT (Clinic)	$0.09	$0.64	-$0.55	T (Transport only^)
Holzman et al (2018)	USA	15/vehicle±	Async.	DOT (Field)	$5.96	$18.26	-$12.30	S, T, F
Krueger et al. (2010)	USA	57	Sync.	DOT (Field)	Incremental		-$41.62	S, T
Lam et al. (2019)	USA	81	Sync.	DOT (Clinic)	$7.84	$10.14	-$2.30	S, T, F
				DOT (Field)	$7.84	$23.78	-$15.94	
		41	Async.	DOT (Clinic)	$6.41	$10.14	-$3.73	
				DOT (Field)	$6.41	$23.78	-$17.36	
Nsengiyumva et al. (2018)	Brazil	NR++ – DS	Sync.	DOT (Clinic)	$7.02	$11.52	-$4.49	S, D, T, F, I, O
		NR++ – DR	Sync.	DOT (Clinic)	$46.13	$51.94	-$5.81	
Nsengiyumva et al. (2023)	Moldova	173 – DS	Async.	DOT (Clinic)	$1.69 ($0.96)	$2.80	-$1.11 (-$1.84)	S, T, F, I
		135 – DR	Async.	DOT (Clinic)	$1.69 ($0.96)	$2.80	-$1.11 (-$1.84)	
	Haiti	87	Async.	DOT (Clinic)	$6.41 ($6.03)	$3.45	$2.96 ($2.58)	
	Philippines	119	Async.	DOT (Clinic)	$2.45 ($1.83)	$0.09	$2.35 ($1.74)	
Siddiqui et al. (2019)	USA	47	Async.	DOT (Field)	$24.32	$42.85	-$18.53	S, T, F
Story et al. (2019)	UK	50§	Sync.	DOT (Clinic)	$13.19	$63.97	-$50.78	S, T, F
Wade et al. (2012)	Australia	47§	Sync**	DOT (Field)	$16.67	$24.92	-$8.25	S, T, F, O

Costs included: S = Staff, D = Drugs and Treatment, T = Travel and supplies, F = Fixed assets and technology, I = Implementation, O = Overhead, P = Patient expenses
DS = Drug susceptible, DR = Drug resistant. Cost in parentheses are calculated with fixed costs annuitized over a 5-year useful life.
§These studies observed costs from 112 patients (Story et al.) and 58 patients (Wade et al.) but modelled clinics with 50 and 47 patients respectively as base cases. These studies also provided multiple scenarios with varying patients per clinic not shown here. 5X/week DOT is shown for Story et al.
^Patients given funds to cover cost of roundtrip to clinic on public transportation
±This study observed costs from 28 patients but modelled a clinic assuming 15 patients per vehicle used in field DOT. ++These patients were a modelled cohort.
**Study considers 5% of patients remaining on DOT even in the DAT case

Table A14: Provider costs for synchronous and asynchronous VOT on a per observation basis – USD based on market exchange rates

					**Provider costs per observation**			
**Study**	**Country**	**Async. pts.**	**Sync. Pts.**	**Setting**	**Asynchronous**	**Synchronous**	**Incremental**	**Costs Included**
Lam et al. (2019)	USA	81	41	**New York City**	**$6.41**	**$7.84**	**-$1.43**	S, T, F
Beeler Asay et al. (2020)	USA	257/year	399/year	All sites	$13.58	$6.64	$6.94	S, T, F, I
		186/year	399/year	**New York City**	**$7.66**	**$6.64**	**$1.02**	
		62/year	-	San Francisco	$8.19	-		
		9/year	-	Rhode Island	$25.40	-		

Costs included: S = Staff, D = Drugs and Treatment, T = Travel and supplies, F = Fixed assets and technology, I = Implementation, O = Overhead, P = Patient expenses
Note: Beeler Asay et al. did not report a site breakdown on the number of patients that made up the combined sample of 63 patients for asynchronous and 57 patients for synchronous VOT. They provided annual patient volumes for each site

Figure A2:: Summary of incremental costs to providers – USD based on market exchange rates

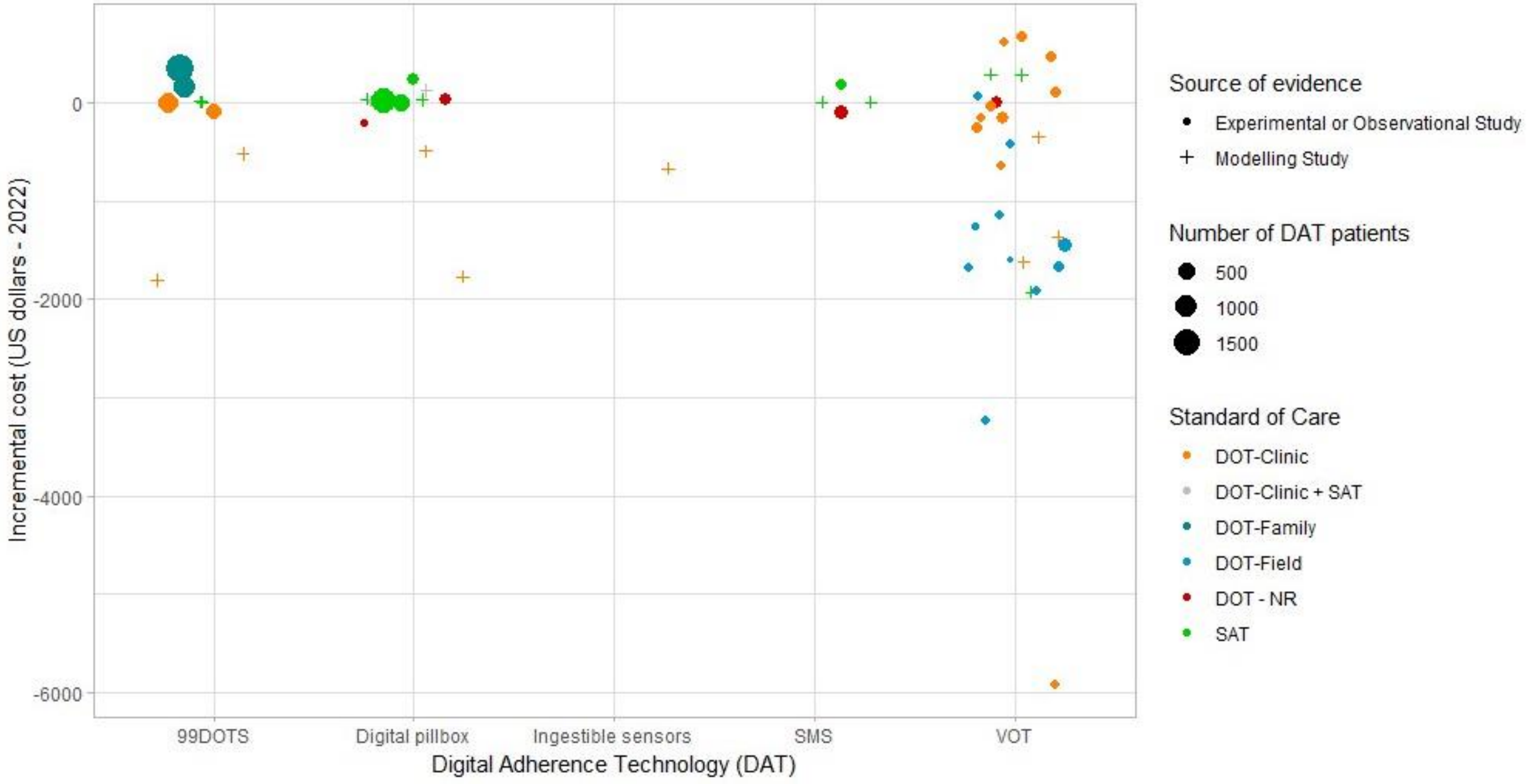

Table A22: CHEERS 2022 Criteria checklist for included studies

**CHEERS 2022 Criteria**	1	2	3	4	5	6	7	8	9	10	11	12	13	14	15	16	17	18	19	20	21	22	23	24	25	26	27	28
Au Yeung et al. (2012)	✓	✓	✓	×	×	×	✓	✓						✓	✓	✓	×			✓	×	✓	✓	×	×	✓	×	✓
Bahrainwala et al. (2020)	✓	✓	✓	×	✓	✓	✓	✓	✓	✓	✓	✓	✓	✓	✓	✓	✓			✓	×	✓	✓	✓	×	✓	✓	✓
Beeler Asay et al. (2020)	✓	✓	✓	×	×	✓	✓	✓	✓					✓	✓	✓	✓	✓		✓	✓	×	✓	×	✓	✓	✓	✓
Broomhead and Mars (2012)	✓	✓	✓	×	✓	✓	×	✓	✓	✓				×	✓	×	✓			×	×	×	✓	×	×	✓	×	✓
Buchman and Cabello (2017)	×	×	✓	×	×	✓	✓	×	✓					✓	×					×	×	×	×	×	×	×	✓	✓
Daftary et al. (2017)			✓	×	✓	✓	✓	×	✓					×	×						×	×	✓		×	✓	✓	✓
Fekadu et al. (2021)	✓	✓	✓	×	✓	✓	✓	✓	✓	✓	✓	✓	✓	✓	✓	✓	✓			✓	×	✓	×	✓	×	✓	✓	✓
Garfein et al. (2018)	×	×	✓	×	✓	×	✓	✓	✓					✓	✓		✓	✓	×	×	✓	×	×	×	✓	✓	✓	✓
Gashu et al. (2021)			×	×	✓	✓	✓	×	✓					✓	×						✓		✓	×	✓	✓	✓	✓
Guo et al. (A) (2020)		✓	✓	×	✓	×	×	×						×	×		×			×	×	×	✓	×	×	×	✓	✓
Guo et al. (B) (2020)		✓	✓	×	✓	✓	✓	×						✓	×		✓				✓	✓	✓		✓	✓	✓	✓
Holzman et al (2018)	×	✓	✓	×	✓	✓	✓	×	×	×				✓	×	×	✓			✓	✓	×	✓	✓	×	✓	✓	✓
Krueger et al. (2010)	×	✓	×	×	×	×	×	×	✓	×				✓	×	✓	✓			×	×	×	✓	×	×	×	✓	×
Lam et al. (2019)	✓	✓	✓	×	×	✓	✓	✓	✓					✓	✓	✓	✓			×	×	✓	✓	×	×	✓	✓	✓
Louwagie et al. (2022)	×	×	✓	✓	✓	×	×	×	✓		✓	✓	✓	✓	✓	×	×			×	×	×	✓	×	×	✓	✓	✓
Manyazewal et al. (2022)	✓	✓	✓	×	✓	×	✓	✓	✓					✓	×		✓			✓	✓	×	×	✓	×	✓	✓	✓
Nsengiyumva et al. (2018)	✓	✓	✓	×	✓	✓	✓	✓	✓	✓	✓	✓	✓	✓	✓	✓	✓	✓	✓	✓	×	✓	✓	✓	×	✓	✓	✓
Nsengiyumva et al. (2023)	✓	✓	✓	×	×	×	×	✓	✓	✓				✓	✓		✓			×	✓	✓	✓	×	×	✓	✓	✓
Ravenscroft et al. (2020)		✓	✓	×	✓	✓	✓	✓						✓	×	✓	×	✓	✓	✓	✓	×	×	✓	✓	✓	✓	✓
Saha et al. (2022)	×	✓	×	×	✓	×	×	×	×	✓	✓	✓	✓	✓	✓	✓	✓			✓	×	×	✓	×	×	✓	✓	✓
Salcedo et al. (2021)	✓	✓	✓	×	✓	✓	✓	✓	✓	×	✓	✓	✓	✓	✓	✓	✓			✓	×	✓	✓	✓	×	✓	✓	✓
Siddiqui et al. (2019)	✓	✓	✓	×	✓	✓	✓	✓	✓					✓	×	✓	×			×	×	×	×	×	✓	✓	×	×
Story et al. (2019)		×		×	✓	×	✓	✓	✓	✓				✓	✓	✓				×	×	✓	✓	×	×	✓	✓	✓
Thompson et al. (2022)	✓	✓	✓	×	×	✓	✓	✓	✓	✓	✓	✓	×	✓	✓	✓	✓			✓	✓	✓	✓	✓	×	✓	✓	✓
Wade et al. (2012)	×	✓	✓	×	✓	×	✓	×	×		✓	✓	✓	✓	×	✓	×			✓	✓	×	✓	✓	✓	✓	✓	✓
Yang et al (2022)	✓	✓	✓	×	✓	✓	✓	✓	✓	✓	✓	✓	✓	✓	✓	✓	✓			✓	×	×	✓	✓	×	✓	✓	✓

Note: Only full text studies evaluated. ✓ Criterion reported. ✓ Criterion not reported. Blank cells are not applicable.

S6 References

1. Maraba N, Orrell C, Chetty-Makkan CM, Velen K, Mukora R, Page-Shipp L, et al. Evaluation of adherence monitoring system using evriMED with a differentiated response compared to standard of care among drug-sensitive TB patients in three provinces in South Africa: a protocol for a cluster randomised control trial. Trials. 2021;22(1):389.
2. Wade VA, Karnon J, Eliott JA, Hiller JE. Home videophones improve direct observation in tuberculosis treatment: a mixed methods evaluation. PLoS One. 2012;7(11):e50155.
3. Thompson RR, Kityamuwesi A, Kuan A, Oyuku D, Tucker A, Ferguson O, et al. Cost and cost-effectiveness of a digital adherence technology for tuberculosis treatment support in Uganda. Value Heal. 2022;
4. Au-Yeung KY, DiCarlo L. Cost comparison of wirelessly vs. directly observed therapy for adherence confirmation in anti-tuberculosis treatment. Int J Tuberc lung Dis. 2012;16(11):1498–504.
5. Bahrainwala L, Knoblauch AM, Andriamiadanarivo A, Diab MM, McKinney J, Small PM, et al. Drones and digital adherence monitoring for community-based tuberculosis control in remote Madagascar: A cost-effectiveness analysis. PLoS One. 2020;15(7):e0235572.
6. Asay GRB, Lam CK, Stewart B, Mangan JM, Romo L, Marks SM, et al. Cost of tuberculosis therapy directly observed on video for health departments and patients in New York City; San Francisco, California; and Rhode Island (2017–2018). Am J Public Health. 2020;110(11):1696.
7. Broomhead S, Mars M. Retrospective return on investment analysis of an electronic treatment adherence device piloted in the Northern Cape Province. Telemed e-Health. 2012;18(1):24–31.
8. Buchman T, Cabello C. A new method to directly observe tuberculosis treatment: Skype observed therapy, a patient-centered approach. J Public Heal Manag Pract. 2017;23(2):175–7.
9. Daftary A, Hirsch-Moverman Y, Kassie GM, Melaku Z, Gadisa T, Saito S, et al. A qualitative evaluation of the acceptability of an interactive voice response system to enhance adherence to isoniazid preventive therapy among people living with HIV in Ethiopia. AIDS Behav. 2017;21(11):3057–67.
10. Fekadu G, Jiang X, Yao J, You JHS. Cost-effectiveness of video-observed therapy for ambulatory management of active tuberculosis during the COVID-19 pandemic in a high-income country. Int J Infect Dis. 2021;113:271–8.
11. Garfein RS, Liu L, Cuevas-Mota J, Collins K, Muñoz F, Catanzaro DG, et al. Tuberculosis treatment monitoring by video directly observed therapy in 5 health districts, California, USA. Emerg Infect Dis. 2018;24(10):1806.
12. Gashu KD, Gelaye KA, Tilahun B. Feasibility, acceptability and challenges of phone reminder system implementation for tuberculosis pill refilling and medication in

Northwest Ethiopia. 2021;

13. Guo P, Qiao W, Sun Y, Liu F, Wang C. Telemedicine technologies and tuberculosis management: a randomized controlled trial. Telemed e-Health. 2020;26(9):1150–6.

14. Guo X, Yang Y, Takiff HE, Zhu M, Ma J, Zhong T, et al. A comprehensive app that improves tuberculosis treatment management through video-observed therapy: usability study. JMIR mHealth uHealth. 2020;8(7):e17658.

15. Holzman SB, Zenilman A, Shah M. Advancing patient-centered care in tuberculosis management: a mixed-methods appraisal of video directly observed therapy. In: Open forum infectious diseases. Oxford University Press US; 2018. p. ofy046.

16. Krueger K, Ruby D, Cooley P, Montoya B, Exarchos A, Djojonegoro BM, et al. Videophone utilization as an alternative to directly observed therapy for tuberculosis. Int J Tuberc Lung Dis. 2010;14(6):779–81.

17. Lam CK, Fluegge K, Macaraig M, Burzynski J. Cost savings associated with video directly observed therapy for treatment of tuberculosis. Int J Tuberc Lung Dis. 2019;23(11):1149–54.

18. Louwagie G, Kanaan M, Morojele NK, Van Zyl A, Moriarty AS, Li J, et al. Effect of a brief motivational interview and text message intervention targeting tobacco smoking, alcohol use and medication adherence to improve tuberculosis treatment outcomes in adult patients with tuberculosis: a multicentre, randomised controlled tri. BMJ Open. 2022;12(2):e056496.

19. Manyazewal T, Woldeamanuel Y, Fekadu A, Holland DP, Marconi VC. Effect of digital medication event reminder and monitor-observed therapy vs standard directly observed therapy on health-related quality of life and catastrophic costs in patients with tuberculosis: a secondary analysis of a randomized clinical trial. JAMA Netw Open. 2022;5(9):e2230509–e2230509.

20. Mukora R, Pelusa R, Gelem A, Xapa Z, Maraba N, Jennings J, et al. Provider costs of using medication monitors and a differentiated care approach to improve TB treatment adherence in South Africa. In: World Conference On Lung Health 2022 Of The International Union Against Tuberculosis And Lung Disease (The Union). Paris; 2022. p. S132.

21. Nsengiyumva NP, Mappin-Kasirer B, Oxlade O, Bastos M, Trajman A, Falzon D, et al. Evaluating the potential costs and impact of digital health technologies for tuberculosis treatment support. Eur Respir J. 2018;52(5).

22. Nsengiyumva NPP, Khan A, Gler MMTS, Lopez M, Marcelo D, Andrews MC, et al. Costs of digital adherence technologies for tuberculosis treatment support. medRxiv. 2023;2003–23.

23. Peng H, Lu W, Xu W. Mobile phone text messaging for promoting adherence to anti-tuberculosis treatment: a community-randomised trial in Jiangsu, China. In: 45th World

Conference on Lung Health of the International Union Against Tuberculosis and Lung Disease (The Union). International Union Against Tuberculosis and Lung Disease (The Union); 2014. p. S213-214.

24. Ravenscroft L, Kettle S, Persian R, Ruda S, Severin L, Doltu S, et al. Video-observed therapy and medication adherence for tuberculosis patients: randomised controlled trial in Moldova. Eur Respir J. 2020;56(2).

25. Saha S, Saxena D, Raval D, Halkarni N, Doshi R, Joshi M, et al. Tuberculosis Monitoring Encouragement Adherence Drive (TMEAD): Toward improving the adherence of the patients with drug-sensitive tuberculosis in Nashik, Maharashtra. Front Public Heal. 2022;10.

26. Salcedo J, Rosales M, Kim JS, Nuno D, Suen S chuan, Chang AH. Cost-effectiveness of artificial intelligence monitoring for active tuberculosis treatment: A modeling study. PLoS One. 2021;16(7):e0254950.

27. Siddiqui S, Wiltz-Beckham D, Fields K, Haynie A, Reed BC, Becker L, et al. Video Directly Observed Therapy for Tuberculosis Treatment at Harris County Public Health: A Cost Analysis and Adherence Assessment. Texas Public Heal J. 2019;71(4).

28. Story A, Aldridge RW, Smith CM, Garber E, Hall J, Ferenando G, et al. Smartphone-enabled video-observed versus directly observed treatment for tuberculosis: a multicentre, analyst-blinded, randomised, controlled superiority trial. Lancet. 2019;393(10177):1216–24.

29. Waswa J, Kityamuwesi A, Thompson R R, Kunihira Tinka L, Nakate A., Namale C, et al. Sustained costs of 99DOTS following implementation in Ugandao Title. In: World Conference On Lung Health 2022 Of The International Union Against Tuberculosis And Lung Disease (The Union). Paris; 2022. p. S109-110.

30. Yang J, Kim HY, Park S, Sentissi I, Green N, Oh BK, et al. Cost-effectiveness of a medication event monitoring system for tuberculosis management in Morocco. PLoS One. 2022;17(4):e0267292.

31. International Monetary Fund. World economic outlook, October 2022 (Database) [Internet]. Washington DC; 2022. Available from: https://www.imf.org/en/Publications/WEO/weo-database/2022/October

32. International Monetary Fund. International Financial Statistics (IFS) - October 2023 [Internet]. Washington DC; 2023. Available from: https://data.imf.org/?sk=388DFA60-1D26-4ADE-B505-A05A558D9A42&sId=1479329132316
